# New Assembly, Reannotation and Analysis of the *Entamoeba histolytica* Genome Reveal New Genomic Features and Protein Content Information

**DOI:** 10.1371/journal.pntd.0000716

**Published:** 2010-06-15

**Authors:** Hernan A. Lorenzi, Daniela Puiu, Jason R. Miller, Lauren M. Brinkac, Paolo Amedeo, Neil Hall, Elisabet V. Caler

**Affiliations:** 1 J. Craig Venter Institute, Rockville, Maryland, United States of America; 2 Center for Bioinformatics and Computational Biology, University of Maryland, College Park, Maryland, United States of America; 3 School of Biological Sciences, University of Liverpool, Liverpool, United Kingdom; New York University School of Medicine, United States of America

## Abstract

**Background:**

In order to maintain genome information accurately and relevantly, original genome annotations need to be updated and evaluated regularly. Manual reannotation of genomes is important as it can significantly reduce the propagation of errors and consequently diminishes the time spent on mistaken research. For this reason, after five years from the initial submission of the *Entamoeba histolytica* draft genome publication, we have re-examined the original 23 Mb assembly and the annotation of the predicted genes.

**Principal Findings:**

The evaluation of the genomic sequence led to the identification of more than one hundred artifactual tandem duplications that were eliminated by re-assembling the genome. The reannotation was done using a combination of manual and automated genome analysis. The new 20 Mb assembly contains 1,496 scaffolds and 8,201 predicted genes, of which 60% are identical to the initial annotation and the remaining 40% underwent structural changes. Functional classification of 60% of the genes was modified based on recent sequence comparisons and new experimental data. We have assigned putative function to 3,788 proteins (46% of the predicted proteome) based on the annotation of predicted gene families, and have identified 58 protein families of five or more members that share no homology with known proteins and thus could be entamoeba specific. Genome analysis also revealed new features such as the presence of segmental duplications of up to 16 kb flanked by inverted repeats, and the tight association of some gene families with transposable elements.

**Significance:**

This new genome annotation and analysis represents a more refined and accurate blueprint of the pathogen genome, and provides an upgraded tool as reference for the study of many important aspects of *E. histolytica* biology, such as genome evolution and pathogenesis.

## Author Summary

*Entamoeba histolytica* is an anaerobic parasitic protozoan that causes amoebic dysentery. The parasites colonize the large intestine, but under some circumstances may invade the intestinal mucosa, enter the bloodstream and lead to the formation of abscesses such amoebic liver abscesses. The draft genome of *E. histolytica*, published in 2005, provided the scientific community with the first comprehensive view of the gene set for this parasite and important tools for elucidating the genetic basis of Entamoeba pathogenicity. Because complete genetic knowledge is critical for drug discovery and potential vaccine development for amoebiases, we have re-examined the original draft genome for *E. histolytica*. We have corrected the sequence assembly, improved the gene predictions and refreshed the functional gene assignments. As a result, this effort has led to a more accurate gene annotation, and the discovery of novel features, such as the presence of genome segmental duplications and the close association of some gene families with transposable elements. We believe that continuing efforts to improve genomic data will undoubtedly help to identify and characterize potential targets for amoebiasis control, as well as to contribute to a better understanding of genome evolution and pathogenesis for this parasite.

## Introduction

Although many infectious diseases receive little attention in today's world, the pathogenic intestinal parasite *E. histolytica* occupies a major place in the list of ignored illnesses. The parasite is the causative agent of amoebiasis, causes a significant level of morbidity and mortality in developing countries, and affects at least 50 million people every year, causing over 100,000 deaths [1]. Yet, a lot is there to be learned about this important protozoan. Genome information allows for better understanding of pathogenic processes and consequently helps improve the prevention, diagnosis, and treatment of the disease. Therefore, accurate and up to date data is fundamental to generate a reliable tool for both research and medical use. The *E. histolytica* genome was automatically annotated and published in 2005 [2]. This draft genome provided the scientific community with the first blueprint of this pathogen, its gene organization and content. However, genome annotation was performed in an automated way, leading to a very raw dataset to work with. Here, in an effort to improve the structural and functional annotation for this organism, we have reviewed, re-assembled and re-annotated the *E. histolytica* genome. The ultimate goal was to generate a high-quality annotation dataset to be used as gold standard by the scientific community and to carry on comparative analysis with the closely related species *Entamoeba dispar* and *Entamoeba invadens*. Using a combination of manual and automated methods we significantly improved the *E. histolytica* assembly. In addition, we generated a new training set of genes for training gene finders, created new gene models and reevaluated and refined previous gene structures based on up to date information, reassessed gene functions, and mapped transposable elements to remove overlapping predicted genes. Here we present an overview of the methods employed for this task and protocols followed, summarizing the contents of the latest data release, with special emphasis on our final assembly and annotation release.

## Methods

### Genome reassembly

Reads were obtained directly from the Sanger Institute and JCVI databases. Reads were filtered based on similarity to an *E. histolytica* plasmid sequence [3] or to tRNA models [4]. Reads were assembled with UMD Overlapper [5] and Celera Assembler [6]. See Text S1 for assembly details. The re-assembled sequence was deposited at the National Center for Biotechnology Information (NCBI) with the accession number AAFB02000000.

### New gene predictions and improvement of gene structures

A set of 20,192 ESTs and 71 full-length cDNAs were downloaded form GenBank. ESTs were assembled and aligned to the newly assembled genome using PASA [7]. A training set consisting of 300 genes supported by 60 full length cDNAs and 240 assembled ESTs was created to train the following gene finders: Genezilla [8], and GlimmerHMM [8]. EVidenceModeler (EVM) [9] was used to generate the new gene dataset, as a weighted consensus of all available evidence, including proteins and conserved protein-domains alignments, cDNAs/ESTs and gene finder output predictions. The new datas[6]et was manually inspected in areas covered by transposable elements (see below). Coding regions shorter than 300 bp supported by no evidence other than Gene Finders were eliminated from the gene dataset. To generate more accurate gene structures in our new dataset, we focused on structural reannotation by improving the accuracy of existing gene models, validating intron/exon boundaries, incorporation of UTRs when available (using PASA), identifying pseudogenes and eliminating spurious genes.

### Repeat finding

First, we created a comprehensive custom database containing all reported *E. histolytica* repetitive elements: LINEs, SINEs, EhERE1 and EhERE2 [10]. Then, we ran RepeatMasker (http://www.repeatmasker.org/) on the current assembly to map and quantify the elements. Regions of the genome that match the repeats were masked to avoid gene prediction on these regions. Any gene predicted on masked regions was removed from the annotation.

### Comparison between original gene annotation (OGA) and new gene annotation (NGA) set

**Figure 1:**
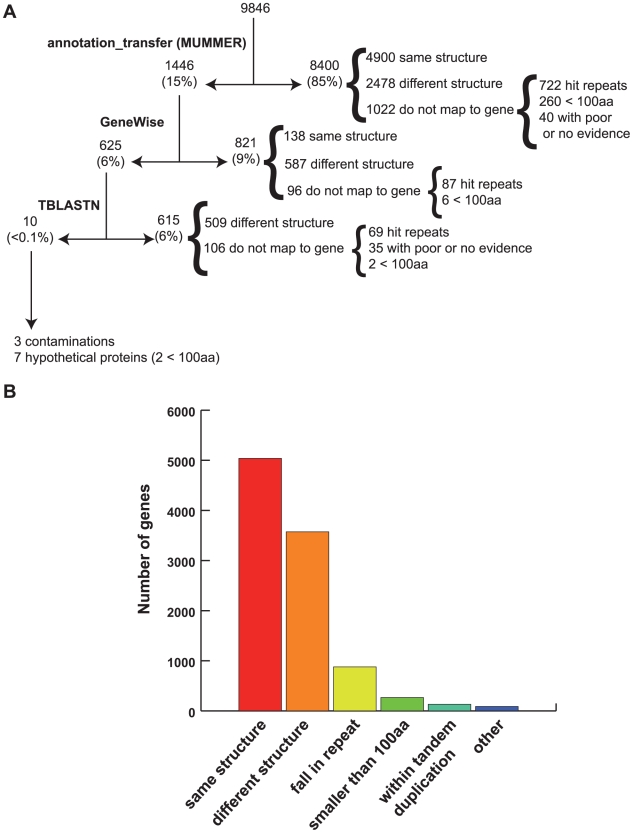
Re-mapping strategy to transfer old annotation A) Steps followed to achieve the full mapping of OGA (9,846 gene models) into the new *E. histolytica* assembly, resulting in NGA (8,201 gene models). B) Mapping of the OGA gene models fell into different categories: genes with perfect map to new assembly (same structure), genes that map to a location but have to be modified (different structure), genes that mapped to a repeat (discarded), genes smaller than 100 amino acids (discarded if they had no evidence), genes that fell within tandem duplications (discarded), and other smaller categories (pseudogenes, truncated genes).

Predicted gene models from the previous assembly were mapped to the new assembly using a combination of methods (Fig. 1). First we identified the correspondence between the scaffolds in the first assembly and the new assembly. Once this correspondence was identified, gene models from the old annotation were mapped onto the new assembly in a multistep fashion. During the first mapping iteration performed with an in-house tool, *annotation_transfer*, based on the software Mummer [11], not all models were transferred as expected due to small sequence variation resulting from a new, independent assembly. In a second mapping round, unmapped genes were aligned to the new assembly using GeneWise [12] an algorithm that combines protein alignment and gene prediction into a single statistical model as a paired Hidden Markov Model (HMM) and provides a gene prediction based on protein homology. Then, genes that failed to map by the previous methods were positioned on the new assembly by tblastn, using a coverage of at least 80% identity, 80% coverage, and an e-value <1×10^−20^. Finally, structural changes between OGA and NGA predictions was assessed using GSAC (Gene Structure Annotation Comparison, unpublished), a JCVI in-house tool that evaluates coordinate differences between two gff3 (generic feature format version 3) files (http://www.sequenceontology.org/gff3.shtml).

### Evaluation of annotation improvement

To evaluate the structural improvement of gene models in the new annotation we selected a dataset of 1024 pairs of genes. Each pair was composed of an OGA and a NGA gene that only map to each other (i.e. they represent the same gene in each annotation) but are structurally different. This dataset was used to perform two types of analyses. First we ran HMM-searches on each pair against the Pfam HMM database and then, we evaluated NGA HMM-searches statistic (e-value, score or number of a particular Pfam domain) compared to their OGA counterparts.

In addition, we performed local blastp searches against our internal non-redundant protein database, PANDA.db (ftp.jcvi.org/pub/data/panda) and identified pairs that shared the same top-hit to run *stretcher*, a global pairwise alignment tool (bioweb2.pasteur.fr/docs/EMBOSS/stretcher.html), between each gene and its corresponding top-hit. Pairs having hits with percent identity below 30% were removed from the results to eliminate false positive hits and results for each pair were analyzed according to their alignment statistics (score, percent identity, percent similarity and percent of gaps) to determine the level of improvement between the annotations. For measuring functional annotation improvement, we estimated the number of genes in the NGA that acquired a descriptive name or an improved name with respect to the OGA only for those genes that did not undergo structural changes to discard functional improvements associated with drastic structural changes, such as incorporation of new exons and changes in coding frame.

### Functional annotation assignments

Gene level searches were performed against protein, domain and profile databases including JCVI in-house non-redundant protein database Panda-AllGroup.niaa, Pfam [13] and TIGRfam [14] HMMs, Prosite [15], and InterPro [16]. In addition, programs to predict membrane localization such as SignalP [17], TMHMM and TargetP [17] were run. After the working gene set had been assigned function, predicted proteins were organized into protein families as previously described [7] with the purpose of refining the annotation in the context of related genes in the genome. Predicted genes were assigned informative names and classified using Gene Ontology (GO) [18]. GO assignments were attributed automatically, based on other assignments from closely related organisms using Pfam2GO, a tool that allows automatic mapping of Pfam hits to GO assignments as well as manually by expert annotators. All assignments were reviewed manually for consistency, on a family based approach, via Manatee, a web-based genome annotation tool that can view, modify, and store annotation for prokaryotic and eukaryotic genomes. Names between OGA and NGA were compared by simple query for corresponding genes to determine the level of change and improvement. Annotation of transporter proteins was performed using TransportDB (http://www.membranetransport.org/) [19].

### Identification of genome duplications

Segmental genome duplications along the *E. histolytica* genome were identified with DAGchainner [20], a program that looks for chains of syntenic genes within complete genome sequences, using default parameters. Briefly, we performed all-vs-all blastp searches using the *E. histolytica* proteome. The blastp output was then filtered out to remove repetitive matches that could potentially contribute noise to the data. Finally, all segmental genome duplications containing five or more duplicated set of genes were further analyzed.

## Results and Discussion

### Characteristic of the new annotation: Improvements to the genome assembly, gene structures and functional assignments

Close examination of the initial assembly of *E. histolytica* strain HM-1:IMSS revealed multiple problems. Sequence analysis using intra-scaffold dot-plots exposed 161 artifactual tandem duplications (Figure S1, panel A) located at the boundaries between neighboring contigs (a contiguous assembled sequence ordered together to form a scaffold). Tandem duplications spanned 364,707 bp of genomic sequence with a median length of 892 bp. In the previous assembly, genes predicted on these regions and on unmasked repetitive regions caused an over-estimation of genes by approximately 18%. Indeed, of the 399 genes located in those regions, 61 hit transposable elements (TEs) or were likely pseudogenes, while most of the remaining 338 coding sequences were artifactually duplicated and so collapsed into 206 individual genes in the new annotation (Figure S1, panel B). A comparative description of the features of the original and the new *E. histolytica* assemblies is summarized in Table 1A.

**Table 1:** Genome statistics and annotation comparison.

A		
**Genome**	**New E. histolytica assembly**	**Old E. histolytica assembly**
Size (bp)	20799072	23361983
GC Content (%)	24.2	24.1
Number of Genes	8201	9985
Mean Gene Length (bp)	1260.9	1170.7
Number of Genes/10 Kbp	3.9	4.3
Longest Gene (bp)	15,210	15,210
Shortest Gene (bp)	147	96
Percent Coding (%)	49.7	50
Percent Genes with Introns (%)	24.4	24.9
**Exons**	**New E. histolytica assembly**	**Old E. histolytica assembly**
Number	10,754	13,176
Mean number per Gene	1.3	1.3
GC Content (%)	28	28.1
Mean Length (bp)	962	886.1
Total Length (bp)	10,340,284	11,675,669
**Introns**	**New E. histolytica assembly**	**Old E. histolytica assembly**
Number	2,553	3191
GC Content (%)	19.3	21.7
Mean Length (bp)	74.1	100
Total Length (bp)	189,260	319,223
**Intergenic Regions**	**New E. histolytica assembly**	**Old E. histolytica assembly**
GC Content (%)	20.5	20
Mean Length (bp)	708.7	823.5

B			
**Annotation**	**OGA**	**NGA**	**NGA-curated genes** *
Genes with EC number	124	1098	604
Genes with GO terms	3106	3468	1843
Number of domains	816	1347	317

A) Comparative genome statistics between the old and current *E. histolytica* genome assemblies. B) Comparative view of EC number, GO terms and domain identification between the old and the new *E. histolytica* annotations. OGA: original genome annotation, NGA: new genome annotation.

*functional annotation was manually reviewed.

The new genome assembly consists of ∼20 Mb of sequence organized into 1,496 scaffolds. To generate a “core” assembly for functional annotation, scaffolds lacking predicted genes were not considered. The resulting core assembly consisted of 818 non-redundant scaffolds with a total of 19,220,345 bp. All scaffolds that were excluded from the core assembly as well as degenerate contigs and singleton reads, although not annotated, were considered to survey the presence or absence of genes when necessary, and all sequences were deposited in GenBank (see Methods).

The results of the new assembly show higher fragmentation and a reduction in genome size with respect of the published assembly. However, our comparative analysis between the two annotations shows that there is no loss of coding information from one assembly to the other.

The new assembly contains 8,201 *de novo* predicted protein coding genes, 1,784 fewer than previously reported for this genome (Table 1) [2]. To determine the origin of these differences and to evaluate changes in gene structure between the original (OGA) and new (NGA) annotation, genes from OGA were mapped onto the new assembly and structural differences were estimated using GSAC (see Methods and Fig. 1A). Mapping results indicated that the main reason for gene number reduction is the elimination of genes within repetitive regions and artifactual tandem duplications, and the removal of genes smaller than 300 bp without any supporting evidence (Fig. 1B). Noteworthy, less than 0.2% of the genes from the original annotation do not map onto the new assembly, despite the fact that the assembly is 2,562,911 bp smaller than the published one. These missing OGA genes contained no supporting evidence and were originally annotated as hypothetical protein coding genes. This analysis also showed that 51% of the OGA genes keep the same structure in the new annotation (same isoform in Fig. 1B), while 36% underwent structural change (different isoform in Fig. 1B).

As part of the curation process, the structure of 740 genes was manually reviewed and curated based on supporting evidence such as ESTs. An important hallmark of this work is the concerted effort from scientists of the Entamoeba community that contributed to the curation of the genome by direct communication with the authors as well as participation via specific workshops held at JCVI.

**Figure 2:**
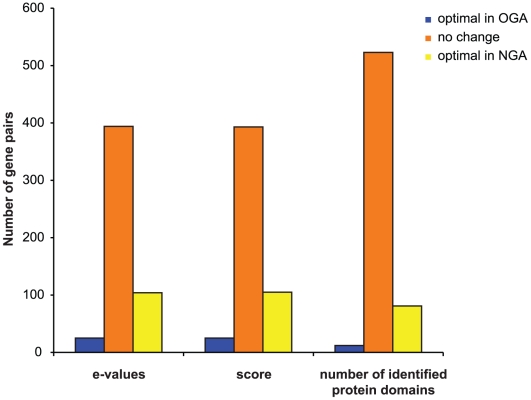
Structural annotation improvement in the new *E. histolytica* assembly. Comparative analysis of Pfam HMM searches statistics between equivalent genes in the old and new annotation. Blue bars, genes that have better statistics/hits in the old annotation compared to the new annotation; orange bars, old and new annotation genes give exactly the same result; yellow bars, number of genes from the new annotation with better statistics/hits compared to their counterparts in the old annotation.

To evaluate whether structural changes in the new annotation reflect an overall improvement of gene structures we selected a group of 1,024 OGA-NGA pairs of genes that map to each other but are structurally different. Then, we ran HMM-searches and global pairwise alignments on each pair of proteins against Pfam HMMs and our PANDA database (see Methods). Finally, we compared the resulting statistics between OGA and NGA peptides from each pair (Fig. 2). These analyses showed that translated products from NGA genes consistently give better hits against Pfam and PANDA databases when compared to OGA genes, demonstrating an overall improvement in gene structures for the new annotation. In those cases where NGA genes gave worse hits compared to their OGA counterparts, we manually inspected and corrected gene structures in the new annotation.

Structural improvements in the new annotation were also reflected by (1) the appearance of new Pfam/TIGRfam domain hits not present in the original protein dataset and (2) the identification of genes coding for additional members of different protein families. Noteworthy, among novel protein domains are a domain typically found in some subunits of several DNA polymerases (PF04042), a domain found in phospholipid methyltransferases (PF04191) and another present in panthotenate kinase proteins (PF03630, see section below). On the other hand, point (2) is very well exemplified by the subunits of the Gal/GalNAc lectins. In *E. histolytica* these lectins are composed of three different subunits: a 170 kDa heavy subunit (Hgl), a 150 kDa intermediate subunit (Igl) and a 31–35 kDa light subunit (Lgl) [21], [22]. In agreement with the current number of *Hgl* and *Lgl* genes in the new annotation, studies of pulse-field gel electrophoresis have shown that there are five *hgl* and six *lgl* genes in the genome [22]. However, only four *Hgl* genes, one of them truncated, and four *Lgl* genes are part of the old dataset.

Particular effort was directed towards the improvement of functional annotation (summarized in Table 1B) by the incorporation of additional 974 enzyme commission (EC) numbers and 531 Pfam/TIGRfam domains. Gene ontology (GO) terms were automatically assigned from Pfam HMM searches refreshing and updating the assignments from InterPro evidence used in the old annotation. The advantage of using hits from Pfam HMM searches is that results can then be filtered not just by e-value but also by trusted cutoff scores, giving a more accurate estimation than InterPro searches and therefore, a more confident GO assignment. In addition to automatic EC number and GO term assignments, functional annotation has been manually curated for 2,130 genes. A total of 3,468 genes have been assigned GO terms, of which 3,216 have a molecular function term. We have distributed the specific terms in a total of 30 molecular function GO-Slim categories (Table S1). No difference was observed in the representation of GO categories in the protein families with respect to that of singletons.

### Classification and function of protein families in *Entamoeba histolytica*

*E. histolytica* predicted proteins were organized into protein families to facilitate the review of their functional annotation, visualizing relationships between proteins and allowing annotators to examine related genes as a group. Our family clustering method produces groups of proteins sharing protein domains conserved across the proteome, and consequently, related biochemical function, as described in Methods [23], [24]. For example, based on our clustering criteria, all proteins containing a single RhoGAP domain (PF00620) fall within the same family irrespectively of their length.

**Figure 3:**
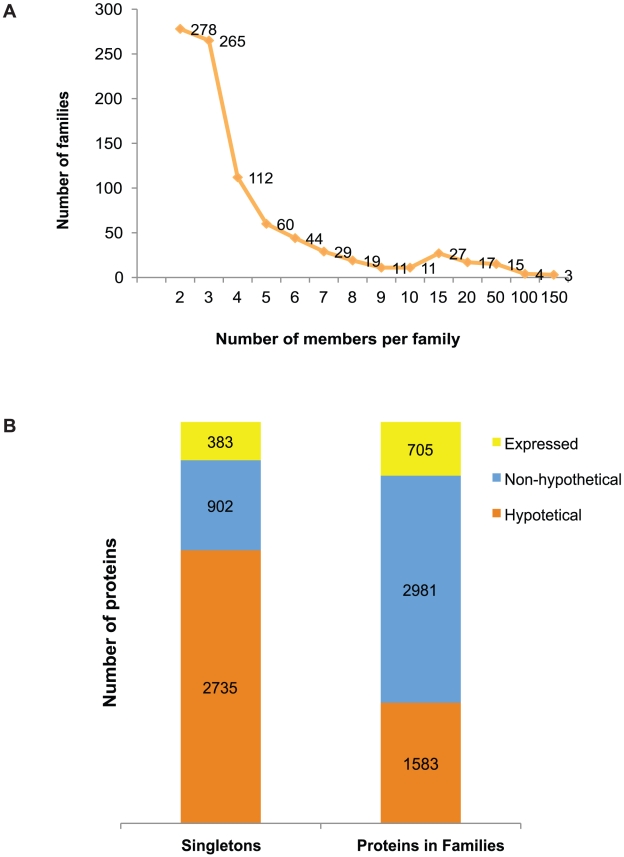
*E. histolytica* protein families. A) Size distribution of protein families. B) Functional assignments in Singletons (proteins not assigned to families) versus Proteins within Families. Hypothetical: predicted hypothetical proteins; Non-hypothetical: predicted proteins with functional assignments; Expressed: predicted proteins with EST (expressed sequence tag) support.

A total of 897 protein families containing 4,564 proteins (56% of the proteome) were identified from the 8,201 predicted polypeptides in the new annotation, leaving 3,637 “orphan” proteins. Among the families, 247 clusters (479 proteins) have no homology to any known Pfam or TIGRfam domain, and harbor potentially novel domains (91 of these families contain five members or more). On average, *E. histolytica* families contain five proteins, ranging from two to 149 members (Fig. 3A). We identified seven families with more than 50 members encoding proteins such as small GTP binding proteins, BspA-like leucine-rich repeat proteins, kinase domain-containing proteins, WD domain-containing proteins, a large family of uncharacterized hypothetical proteins, a RNA recognition motif domain-containing protein family and a RhoGAP domain-containing protein family (see Table S2 for the complete list of families).

Interestingly, a number of protein families appear to be physically linked to transposable elements. Table 2 shows the top 27 families that present this type of association (for the entire repertoire of genes see Table S3). For example, a cluster of 31 members of the Hsp70 protein family appears associated 30% of the time with TEs within 1 kb of the gene context. Hsp70 proteins are molecular chaperones that assist a large variety of protein folding processes in the cell by the transient association between their substrate-binding domain and the short hydrophobic peptide segments present in their target proteins. Hsp70 s are highly conserved and are known to be induced by a variety of stresses [25]. It has been previously reported that multiple natural TE insertions in *Drosophila* reduce the level of expression of *hsp70* genes by insertion nearby gene promoter regions [26]. The characteristics of the *hsp70* promoter in the fly may make it a suitable target for transposition leading to the generation of novel alleles. In this sense, TEs could be playing an adaptive role in microevolution by gene amplification and also manipulating the expression of genes critical for the parasite fitness [27].

**Table 2:** *Entamoeba histolytica* protein families showing high association with repetitive elements.

Family ID*	Protein family name	Number of associated elements	Number of genes in Family	Percentage of Association
**238**	hypothetical protein	5	5	100%
**133**	hypothetical protein	7	7	100%
**64**	hypothetical protein	10	10	100%
**145**	hypothetical protein	5	6	83%
**52**	hypothetical protein	4	5	80%
**236**	cystein protease family	4	5	80%
**66**	hypothetical protein	6	8	75%
**42**	hypothetical protein, conserved	11	15	73%
**157**	Gal/Gal/Nac lectin complex family	4	6	66%
**87/29/274**	AIG1 family protein	18	29	62%
**77**	regulator of nonsense transcripts family	6	10	60%
**93**	hypothetical protein	5	9	55%
**111**	hypothetical protein	4	8	50%
**15**	hypothetical protein	12	29	41%
**67**	hypothetical protein	4	11	36%
**2**	BspA-like family protein	41	114	35%
**12**	HSP 70 family	11	31	35%
**63**	peroxiredoxin family protein	4	12	33%
**54**	hypothetical protein	4	13	30%
**41**	cystein protease family	4	14	28%
**32**	DEAD/DEAH-box helicase family protein	5	18	27%
**9**	kinase family protein	9	39	23%
**19**	zinc-finger domain containing protein	6	26	23%
**8**	hypothetical protein	9	38	23%
**5**	hypothetical protein, conserved	13	61	21%
**24**	kinase family protein	4	20	20%
**13**	LRR repeat containing protein	5	29	17%

*Only families with at least five proteins and showing more than 15% association are shown.

Another family showing a high correlation with transposable elements is the large BspA-like surface protein family [28], [29]. Initially, Davis *et al*. identified 89 genes coding for BspA-like proteins in the genome of *E. histolytica*, containing a leucine-rich repeat motif (LRRs). LRRs serve as recognition motifs for surface proteins in bacteria and other eukaryotes [30] and have been shown to be involved in binding to fibronectin. *E. histolytica* BspA-like proteins have unique LRR-like repeats that resemble, to certain extent, to the *Treponema pallidum* LLRs (LrrA proteins) [28], that appear to have a role in attachment and penetration to host tissues [31], suggesting they may be involved in attachment to the host cells. Our analysis identified 116 BspA-like genes in the genome, 41 of them associated with transposable elements. The core domain of the BspA-like proteins contains 23 amino acids with the consensus P[T/S][T/S][V/I/L]xx[I/L]GxxCFxxCxxLxx[I/L]x[I/L], and these units form tandem blocks that can contain two or more core motifs represented from 1 to 21 times in a single molecule, leading to a great variability in the protein length in the family. Most of the proteins in the family contain a novel 50 amino acids N-terminal domain that is preserved in 85 members of this cluster. A closer examination of those genes encoding proteins lacking the N-terminal domain showed they are probably truncated by the insertion of transposable elements, primarily SINE and LINE elements at their 5′ end. BspA-like proteins are located on the surface of *E. histolytica* [28] however no classic membrane-targeting signal is present in the proteins. Therefore, it is tempting to speculate that the conserved N-terminal domain of these proteins might function as either an export signal or serve as a membrane-anchor domain or that export involves a non-classical transport mechanism, independent of the ER–Golgi pathway, similar to those that have been detected in yeast and mammalian cells [32]. Details on the motifs and domain structure are shown in Figure S2.

A third worthy of note family associated with TEs is the AIG family of proteins, comprising 29 members distributed in 3 clusters, of which 18 genes are in close proximity to repetitive elements (Table 2). AIG1 proteins are associated with resistance to bacteria [33]. Interestingly, comparative gene expression studies have shown that AIG1 proteins as well as heat shock proteins have significantly reduced expression levels in *E. dispar* [34], when compared to *E. histolytica*. This observation leads us to speculate that transposable elements inserted in the neighborhood of these genes could lead to the enhanced expression of these genes and ultimately could be related to the increased virulence. Indeed we have previously shown that LINEs and SINEs are involved in genome rearrangements driving in consequence genomic evolution [10]. It is tempting to speculate that the amplification of the AIG family was mediated by the close association of TEs, but the observation that non-virulent *E. dispar* contains the same number of genes without the TE association seems to indicate that this is not the case. We are currently analyzing all gene family/transposable element associations in the context of comparative genomics with other Entamoeba species (manuscript in preparation).

Close examination of the functional annotation of protein families and singleton proteins revealed that a total of 2,981 (65%) genes within the families were annotated as encoding proteins with putative functions and 1,583 genes are hypothetical proteins (34%, Fig. 3B). Of a total of 1,088 genes that have EST support in the whole genome, 705 are genes within protein families. In contrast, singletons had a larger proportion of hypothetical genes (76%) and a smaller portion of genes with a known or putative function (24%), and half the number of genes supported by EST evidence (383).

### Segmental genome duplications

**Figure 4:**
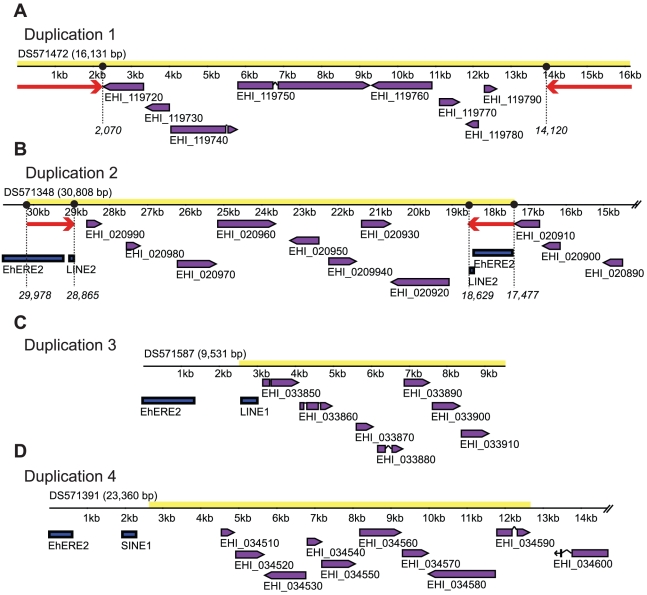
*Entamoeba histolytica* segmental genome duplications. A) D1-type duplications flanked by unique 2.3 kb inverted repeats (IR), B) D2-type duplications flanked by EhERE1/EhLINE2- derived 1.2 kb IRs, C) D3-type duplications usually associated to EhLINE1, but lacking IRs, and D) D4-type duplications present in the vicinity of TE elements, and lacking IRs. Inverted red arrows: IRs; purple boxes: open reading frames; blue boxes: repetitive elements; DS identifiers correspond to GenBank accession numbers for the corresponding scaffolds.

As mentioned above, about 20% of the *E. histolytica* genome consists of transposable elements. These repeats show a tendency to insert close to each other forming large TE clusters. We have previously shown that these repeat clusters are frequently found at syntenic breakpoints between *E. histolytica* and *E. dispar* suggesting that they could contribute to parasite genome instability and, consequently, to the evolution of these species [10]. It is also possible that the highly repetitive nature of this genome led to genome duplications. In order to evaluate this possibility we analyzed the presence of additional rearrangements within the genome by searching for segmental duplications using DAGchainer as explained in Methods [20]. We observed the presence of four different types of segmental duplications, named D1-D4, spanning seven to ten genes each (Fig. 4).

The first duplication (D1, Fig. 4A) spans a 16.6 kb region containing up to 8 hypothetical protein coding genes. These duplications are approximately 94% identical at the nucleotide level. All D1-type duplications are flanked by 2.3 kb inverted repeats (IR) not found in the rest of the genome. Nucleotide composition analysis revealed that D1-IRs are highly AT-rich (84.3%) compared to the average content of those regions 71.4% and they are 95% identical at the nucleotide level. A genome wide survey of D1-duplications led to the identification of four complete and two partial copies of this element in the genome. It is interesting to mention that all the scaffolds containing the four complete duplications have similar sizes (16.6 kb on average) and are spanned almost in their entire length by their respective segmental duplications. The two partial D1-duplications are located in shorter scaffolds of 14.4 kb and 6.6 kb, respectively.

The second duplication (D2, Fig. 4B) is 12.5 kb long and contains up to eight duplicated hypothetical protein coding genes depending on the duplication. Comparative analysis showed that these duplications are more than 80% identical at the nucleotide level with an average of 92%. Similar to D1-type duplications, D2 are frequently flanked by 1.2 kb IRs, composed of two fragments derived from the TEs EhERE1 and EhLINE2. D2-IRs share 92.6% identity at the nucleotide level and are also very AT-rich (85%AT). The organization of the duplications is not conserved in all copies across the genome, with some copies flanked by IRs composed of either EhERE1 or EhLINE2 fragments, while in others we could not identify any IR.

D3-type duplications are 7.4 kb long and 83% identical at the nucleotide level. Although frequently found nearby TEs (mostly EhLINE1), none of the eight identified genome duplications are flanked by IRs as D1- and D2-type duplications. D3 presents a very unique gene content that suggest that the segment could present a unique functionality, represented in Fig. 4C. A total of seven protein coding genes are arranged in the same orientation, and include a putative serine-threonine kinase similar to ARK1, a human protein that participates in cell cycle regulation; an endonuclease V domain-containing protein coding gene potentially involved in DNA repair; a putative secreted hypothetical protein coding gene; a tandem duplicated gene coding for a putative protein containing a type-1 glutamine amido transferase-like domain and a GDSL-like lipase/acylhydrolase domain-containing protein coding gene. Interestingly, D3-type duplications are found at or in close proximity to the end of scaffolds, and therefore, they could potentially be located at subtelomeric regions. However, in spite of a thorough analysis we could not identify any repetitive telomeric/subtelomeric motif in these regions.

Lastly, the 10 kb long D4 (Fig. 4D) shares more than 85% identity at the nucleotide level and spans up to 9 hypothetical protein coding and one putative dUTP hydrolase-coding genes. Most D4-type duplications have TEs inserted nearby, but no flanking IRs were identified.

The presence of these duplications is not likely to be an artifact of the assembly due to the fact that they are also appear duplicated in *E. dispar*. It is possible that some of these duplications, that in some cases span full scaffolds represent different copies of one of the several extrachromosomal elements known to exist in Entamoeba species, as described by Dhar *et al* [35].

### New features

Our work has led to the identification of 460 novel putative protein coding genes not present in the OGA, 16% of which have some functional annotation. One of these genes codes for a putative pantothenate kinase (EHI_183060) the first enzyme in the biosynthesis of coenzyme A from pantothenate. Although the coding genomic region was present in the original assembly, the gene had not been predicted and therefore, it was missing from the previous annotation. Only the enzymes phophopantothenoyl-cysteine decarboxilase (EC 4.1.1.36), phosphopantothenoyl-cysteine synthase (EC 6.3.2.5), and dephospho-CoA kinase (EC 2.7.1.24), responsible for the second, third and last of the five consecutive enzymatic reactions, had been previously identified in the OGA (EHI_164490, EHI_092330, EHI_040840). However, the lack of candidate enzymes for the remaining two biochemical reactions of this pathway raised the question whether *E. histolytica* can synthesize coenzyme A from pantothenate [36]. Our *de novo* gene prediction for a putative pantothenate kinase plus the identification of a candidate gene for the forth step of this pathway, a putative pantetheine-phosphate adenylyltransferase (EC 2.7.7.3), indicates that the whole set of metabolic reactions required to synthesize coenzyme A from pantothenate is present in this amoeba. Interestingly, the enzymes that participate in this pathway resemble those from eubacteria but not higher eukaryotes. Indeed, the second and third sets of reactions are catalyzed by a single enzyme present in two copies (EHI_164490, EHI_092330), while the fourth and fifth steps are carried out by independent enzymes, EHI_006680 and EHI_040840, respectively. In higher eukaryotes the last two reactions are carried out by the same enzyme [37].

Another gene not present in the OGA (EHI_141410) codes for a protein with a predicted molecular weight of 44.6 kDa similar to subunit p50 of the DNA polymerase delta, a key enzyme for chromosomal DNA replication in higher eukaryotes. In mammals, it has been shown that p50 is tightly associated with p125, the catalytic subunit of these types of DNA polymerases. Accordingly, a gene coding for a putative 124.4 kDa catalytic subunit of the DNA polymerase delta (EHI_006690), is also present in the NGA. These results are in agreement with a previous work showing that the sensitivity to different inhibitors of the DNA polymerase activity of *E. histolytica* resembles that of mammalian DNA alpha, delta and epsilon polymerases [38].

In addition, a gene coding for a protein containing a Yos1-like Pfam domain is also absent from OGA (EHI_178640). This putative protein has similarity to other members of the Yos1 family, involved in protein transport between the endoplasmic reticulum and the Golgi apparatus [39].

Comparative analysis between the two annotation datasets also allowed us to identify genes present in their complete form in NGA but truncated in OGA. Example of these genes are two copies of a gene coding for a putative pyridine nucleotide transhydrogenase, EHI_055400 and EHI_014030, the latter identical to a gene previously cloned by Clark *et al*. [40], which exists as a single truncated copy in the OGA. Another example is a 605 bp gene coding for a putative phospholipid methyltransferase protein (EHI_153710) similar to *Schizosaccharomyces pombe* cho1 (35% identity; e-value  = 4×10^−21^), an enzyme that participates in the synthesis of phosphatidylcholine via the methylation of phosphatidylethanolamine. A coding sequence containing only the last 222 bp of this gene is present in the OGA.

### Final remarks

Our reannotation effort has focused mostly on the improvement of the assembly and the gene content and structure of the *E. histolytica* genome. The new assembly, annotation and analysis of the genome has incorporated many updates and enhancements to the structural and functional assignments of the original gene predictions, including an improved assembly, removal of spurious genes, improved gene structures and functional assignments, and generation of gene families.

Regardless of the advancement of the computational methods and of the exponentially growing amount of data that could be used for automated genome annotation, only experimental evidence from expression data will conclusively validate the accuracy of computationally assigned functions done at the genome-wide level. Nevertheless, in order to provide a sound bases to drive research, genome annotations have to be maintained and revised, either by expert annotators in the field and/or community involvement. Additional sequence information will allow the further refinement of gene structures and a deeper understanding of the genome architecture, while the functional annotation will be enriched both by the availability of new experimental data and from expression and other kinds of analyses to characterize each gene and its function fully.

This reannotation effort will be the base for the future analysis and annotation of new *E. histolytica* genomes from patient isolates, a project recently approved under the NIAID supported program Genome Sequence Centers for Infectious Disease, GSCID (http://gsc.jcvi.org/).

## Supporting Information

Figure S1 Example of artifactual tandem duplications identified in the old *Entamoeba histolytica* assembly. A) Dot-plot analysis of scaffold Scaffold_00115 reveals the presence of two tandem duplications spanning contig junctions. Numbers on top and on the left of the dot-plot indicate positions in base pairs along the scaffold. Green lines represent contigs and tandem duplications 1 and 2 within contigs are denoted by yellow and light-blue boxes, respectively. Contig GenbBank accession numbers are shown on the left. B) Schematic representation of the resolution of the artifactual tandem duplication 1 in the new assembly. Old (Scaffold_00115) and new (DS571349) scaffolds are represented as black horizontal lines. Colored boxes above or below the scaffold represent genes on the forward or reverse strand, respectively. Grey areas represent tandem duplicated regions and how they are resolved on the new assembly. Numbers indicate positions in base pairs within scaffolds. Scaffold sequence truncations are represented by double back-slashes. Old and new locus tags are depicted above and below genes, respectively.
(0.43 MB EPS)

Figure S2 Domain composition of BspA-like family proteins. A) LOGO for consensus N-terminal conserved domain; B) LOGO for consensus LRR-like repetitive domain; C) schematic representation of typical BspA-like family proteins showing different number of domains; D) size distribution chart showing the great variation in size in the family members due to the different number of domain units. Domain logos were created using WebLogo.
(1.44 MB EPS)

Table S1 Distribution of *Entamoeba histolytica* genes into GO slim categories. The table shows the number of *E. histolytica* genes associated to each GO slim category we selected for this genome. Column 1, GO identifier; column 2, number of genes that share that GO slim; column 3, GO identifier definition.
(0.05 MB DOC)

Table S2 *Entamoeba histolytica*-specific families. Table S2 lists families of *Entamoeba histolytica* proteins that do not share homology with any other organisms but *E. histolytica*. Some of the families may share a degree of homology to other closely related *Entamoeba* species. Column 1, Family ID corresponds to the specific identifier for each group; column 2, product name assigned to each gene; column 3, public locus name that represents the stable identifier, searchable in GenBank.
(0.76 MB DOC)

Table S3 *Entamoeba histolytica* genes associated to Repetitive elements. Table S3 provides the complete list of *E. histolytica* protein families that show a close association (within 1 kb upstream or downstream) with transposable elements.
(0.54 MB DOC)

Text S1 Assembly supplement.
(0.05 MB DOC)
