# Supplementary material for: New Assembly, Reannotation and Analysis of the Entamoeba histolytica Genome Reveal New Genomic Features and Protein Content Information
Source: PLoS Negl Trop Dis. 2010 Jun 15;4(6):e716. doi: 10.1371/journal.pntd.0000716 (PMC2886108; doi:10.1371/journal.pntd.0000716)
Supplement: Table S1 — Distribution of Entamoeba histolytica genes into GO slim categories. The table shows the number of E. histolytica genes associated to each GO slim category we selected for this genome. Column 1, GO identifier; column 2, number of genes that share that GO slim; column 3, GO identifier definition. (0.05 MB DOC) [file pntd.0000716.s003.doc]

| Table S1. Distribution of *E. histolytica* genes into GO slim categories | | |
| --- | --- | --- |
| GO Term | Number of genes in category | GO definition |
| GO:0000166 | 739 | nucleotide binding |
| GO:0003674 | 327 | molecular function |
| GO:0003676 | 164 | nucleic acid binding |
| GO:0003677 | 147 | DNA binding |
| GO:0003682 | 4 | chromatin binding |
| GO:0003700 | 20 | transcription factor activity |
| GO:0003723 | 97 | RNA binding |
| GO:0003774 | 9 | motor activity |
| GO:0003779 | 38 | actin binding |
| GO:0003824 | 573 | catalytic activity |
| GO:0004518 | 55 | nuclease activity |
| GO:0004672 | 369 | protein kinase activity |
| GO:0004721 | 129 | phosphoprotein phosphatase activity |
| GO:0004871 | 3 | signal transducer activity |
| GO:0004872 | 2 | receptor activity |
| GO:0005198 | 209 | structural molecule activity |
| GO:0005215 | 133 | transporter activity |
| GO:0005216 | 4 | ion channel activity |
| GO:0005488 | 218 | binding |
| GO:0005509 | 41 | calcium ion binding |
| GO:0005515 | 217 | protein binding |
| GO:0008135 | 26 | translation factor activity, nucleic acid binding |
| GO:0008233 | 119 | peptidase activity |
| GO:0008289 | 13 | lipid binding |
| GO:0016209 | 18 | antioxidant activity |
| GO:0016301 | 134 | kinase activity |
| GO:0016740 | 292 | transferase activity |
| GO:0016787 | 539 | hydrolase activity |
| GO:0030234 | 225 | enzyme regulator activity |
| GO:0030246 | 6 | carbohydrate binding |
| GO:0030528 | 24 | transcription regulator activity |
