# Supplementary material for: New Assembly, Reannotation and Analysis of the Entamoeba histolytica Genome Reveal New Genomic Features and Protein Content Information
Source: PLoS Negl Trop Dis. 2010 Jun 15;4(6):e716. doi: 10.1371/journal.pntd.0000716 (PMC2886108; doi:10.1371/journal.pntd.0000716)
Supplement: Table S2 — Entamoeba histolytica-specific families. Table S2 lists families of Entamoeba histolytica proteins that do not share homology with any other organisms but E. histolytica. Some of the families may share a degree of homology to other closely related Entamoeba species. Column 1, Family ID corresponds to the specific identifier for each group; column 2, product name assigned to each gene; column 3, public locus name that represents the stable identifier, searchable in GenBank. (0.76 MB DOC) [file pntd.0000716.s004.doc]

| Table S2: *E. histolytica* specific families. | | |
| --- | --- | --- |
| Family ID | Protein name | Public locus (GenBank) |
| 10 | hypothetical protein | EHI_003950 |
| 10 | hypothetical protein | EHI_004520 |
| 10 | hypothetical protein | EHI_011400 |
| 10 | hypothetical protein | EHI_012250 |
| 10 | hypothetical protein | EHI_029390 |
| 10 | hypothetical protein | EHI_033010 |
| 10 | hypothetical protein | EHI_038140 |
| 10 | hypothetical protein | EHI_038820 |
| 10 | hypothetical protein | EHI_046380 |
| 10 | hypothetical protein | EHI_050560 |
| 10 | hypothetical protein | EHI_052760 |
| 10 | hypothetical protein | EHI_065650 |
| 10 | hypothetical protein | EHI_069090 |
| 10 | hypothetical protein | EHI_075720 |
| 10 | hypothetical protein | EHI_081240 |
| 10 | hypothetical protein | EHI_082210 |
| 10 | hypothetical protein | EHI_092180 |
| 10 | hypothetical protein | EHI_096610 |
| 10 | hypothetical protein | EHI_096270 |
| 10 | hypothetical protein | EHI_102030 |
| 10 | hypothetical protein | EHI_105350 |
| 10 | hypothetical protein | EHI_118650 |
| 10 | hypothetical protein | EHI_129830 |
| 10 | hypothetical protein | EHI_129920 |
| 10 | hypothetical protein | EHI_129930 |
| 10 | hypothetical protein | EHI_132390 |
| 10 | hypothetical protein | EHI_137050 |
| 10 | hypothetical protein | EHI_137060 |
| 10 | hypothetical protein | EHI_137260 |
| 10 | hypothetical protein | EHI_151750 |
| 10 | hypothetical protein | EHI_160680 |
| 10 | hypothetical protein | EHI_163490 |
| 10 | hypothetical protein | EHI_165300 |
| 10 | hypothetical protein | EHI_166460 |
| 10 | hypothetical protein | EHI_184790 |
| 10 | hypothetical protein | EHI_187030 |
| 10 | hypothetical protein | EHI_192550 |
| 10 | hypothetical protein | EHI_192580 |
| 100 | hypothetical protein | EHI_034540 |
| 100 | hypothetical protein | EHI_059290 |
| 100 | hypothetical protein | EHI_190320 |
| 100 | hypothetical protein | EHI_193680 |
| 100 | hypothetical protein | EHI_018260 |
| 100 | hypothetical protein | EHI_020720 |
| 100 | hypothetical protein | EHI_028780 |
| 100 | hypothetical protein | EHI_196750 |
| 102 | hypothetical protein | EHI_015240 |
| 102 | hypothetical protein | EHI_020970 |
| 102 | hypothetical protein | EHI_074560 |
| 102 | hypothetical protein | EHI_089440 |
| 102 | hypothetical protein | EHI_095320 |
| 102 | hypothetical protein | EHI_095460 |
| 102 | hypothetical protein | EHI_145280 |
| 102 | hypothetical protein | EHI_154710 |
| 104 | hypothetical protein | EHI_106260 |
| 104 | hypothetical protein | EHI_179090 |
| 104 | hypothetical protein, conserved | EHI_017610 |
| 104 | hypothetical protein, conserved | EHI_114220 |
| 104 | hypothetical protein, conserved | EHI_117860 |
| 104 | hypothetical protein, conserved | EHI_124390 |
| 104 | hypothetical protein, conserved | EHI_147440 |
| 104 | hypothetical protein, conserved | EHI_184500 |
| 108 | hypothetical protein | EHI_002250 |
| 108 | hypothetical protein | EHI_034580 |
| 108 | hypothetical protein | EHI_037710 |
| 108 | hypothetical protein | EHI_074500 |
| 108 | hypothetical protein | EHI_164170 |
| 108 | hypothetical protein | EHI_196730 |
| 108 | hypothetical protein | EHI_144150 |
| 109 | hypothetical protein | EHI_001900 |
| 109 | hypothetical protein | EHI_008700 |
| 109 | hypothetical protein | EHI_035100 |
| 109 | hypothetical protein | EHI_050880 |
| 109 | hypothetical protein | EHI_062970 |
| 109 | hypothetical protein | EHI_139300 |
| 109 | hypothetical protein | EHI_156220 |
| 109 | hypothetical protein | EHI_003030 |
| 11 | hypothetical protein | EHI_006000 |
| 11 | hypothetical protein | EHI_008180 |
| 11 | hypothetical protein | EHI_023280 |
| 11 | hypothetical protein | EHI_024400 |
| 11 | hypothetical protein | EHI_029010 |
| 11 | hypothetical protein | EHI_031210 |
| 11 | hypothetical protein | EHI_038150 |
| 11 | hypothetical protein | EHI_039720 |
| 11 | hypothetical protein | EHI_045580 |
| 11 | hypothetical protein | EHI_050490 |
| 11 | hypothetical protein | EHI_065730 |
| 11 | hypothetical protein | EHI_076020 |
| 11 | hypothetical protein | EHI_079980 |
| 11 | hypothetical protein | EHI_086020 |
| 11 | hypothetical protein | EHI_086000 |
| 11 | hypothetical protein | EHI_106270 |
| 11 | hypothetical protein | EHI_123700 |
| 11 | hypothetical protein | EHI_126200 |
| 11 | hypothetical protein | EHI_127310 |
| 11 | hypothetical protein | EHI_137010 |
| 11 | hypothetical protein | EHI_137270 |
| 11 | hypothetical protein | EHI_139400 |
| 11 | hypothetical protein | EHI_152030 |
| 11 | hypothetical protein | EHI_153020 |
| 11 | hypothetical protein | EHI_152780 |
| 11 | hypothetical protein | EHI_156090 |
| 11 | hypothetical protein | EHI_159490 |
| 11 | hypothetical protein | EHI_165190 |
| 11 | hypothetical protein | EHI_167690 |
| 11 | hypothetical protein | EHI_168250 |
| 11 | hypothetical protein | EHI_182940 |
| 11 | hypothetical protein | EHI_187210 |
| 11 | hypothetical protein | EHI_189440 |
| 11 | hypothetical protein | EHI_056610 |
| 110 | hypothetical protein | EHI_007750 |
| 110 | hypothetical protein | EHI_010340 |
| 110 | hypothetical protein | EHI_050800 |
| 110 | hypothetical protein | EHI_093970 |
| 110 | hypothetical protein | EHI_127030 |
| 110 | hypothetical protein | EHI_150100 |
| 110 | hypothetical protein | EHI_152220 |
| 110 | hypothetical protein | EHI_182770 |
| 111 | hypothetical protein | EHI_020990 |
| 111 | hypothetical protein | EHI_078540 |
| 111 | hypothetical protein | EHI_089460 |
| 111 | hypothetical protein | EHI_095300 |
| 111 | hypothetical protein | EHI_095440 |
| 111 | hypothetical protein | EHI_130460 |
| 111 | hypothetical protein | EHI_130470 |
| 111 | hypothetical protein | EHI_154690 |
| 112 | hypothetical protein | EHI_020950 |
| 112 | hypothetical protein | EHI_062330 |
| 112 | hypothetical protein | EHI_074590 |
| 112 | hypothetical protein | EHI_089420 |
| 112 | hypothetical protein | EHI_095480 |
| 112 | hypothetical protein | EHI_145320 |
| 112 | hypothetical protein | EHI_154730 |
| 112 | hypothetical protein | EHI_130520 |
| 113 | hypothetical protein | EHI_000540 |
| 113 | hypothetical protein | EHI_014020 |
| 113 | hypothetical protein | EHI_052730 |
| 113 | hypothetical protein | EHI_087700 |
| 113 | hypothetical protein | EHI_103380 |
| 113 | unknown protein, putative | EHI_169930 |
| 113 | transcriptional regulator cudA, putative | EHI_127960 |
| 114 | hypothetical protein | EHI_054940 |
| 114 | mucin-5AC, putative | EHI_176380 |
| 114 | hypothetical protein | EHI_189750 |
| 114 | G-box-binding factor, putative | EHI_158940 |
| 114 | mucin-like protein 1 precursor, putative | EHI_041680 |
| 114 | serine-rich 25 kDa antigen protein, putative | EHI_112490 |
| 121 | hypothetical protein | EHI_020940 |
| 121 | hypothetical protein | EHI_062340 |
| 121 | hypothetical protein | EHI_074600 |
| 121 | hypothetical protein | EHI_095490 |
| 121 | hypothetical protein | EHI_130530 |
| 121 | hypothetical protein | EHI_145330 |
| 121 | hypothetical protein | EHI_154740 |
| 126 | hypothetical protein | EHI_097050 |
| 126 | hypothetical protein | EHI_116050 |
| 126 | hypothetical protein | EHI_126530 |
| 126 | hypothetical protein | EHI_147990 |
| 126 | hypothetical protein | EHI_171450 |
| 126 | hypothetical protein | EHI_197140 |
| 126 | hypothetical protein | EHI_112390 |
| 132 | hypothetical protein | EHI_004550 |
| 132 | hypothetical protein | EHI_010570 |
| 132 | hypothetical protein | EHI_086690 |
| 132 | hypothetical protein | EHI_103860 |
| 132 | hypothetical protein | EHI_189930 |
| 132 | TolA protein, putative | EHI_159620 |
| 132 | TolA-like protein, putative | EHI_052780 |
| 133 | hypothetical protein | EHI_017900 |
| 133 | hypothetical protein | EHI_057810 |
| 133 | hypothetical protein | EHI_087690 |
| 133 | hypothetical protein | EHI_109270 |
| 133 | hypothetical protein | EHI_191520 |
| 133 | hypothetical protein | EHI_135600 |
| 133 | hypothetical protein | EHI_190420 |
| 143 | hypothetical protein, conserved | EHI_006940 |
| 143 | hypothetical protein, conserved | EHI_049600 |
| 143 | hypothetical protein, conserved | EHI_092680 |
| 143 | hypothetical protein, conserved | EHI_131130 |
| 143 | hypothetical protein, conserved | EHI_136740 |
| 143 | hypothetical protein, conserved | EHI_152470 |
| 145 | hypothetical protein | EHI_065890 |
| 145 | hypothetical protein | EHI_091350 |
| 145 | hypothetical protein | EHI_143940 |
| 145 | hypothetical protein | EHI_183210 |
| 145 | hypothetical protein | EHI_011560 |
| 145 | hypothetical protein | EHI_075310 |
| 146 | hypothetical protein | EHI_001780 |
| 146 | hypothetical protein | EHI_009620 |
| 146 | hypothetical protein | EHI_072530 |
| 146 | hypothetical protein | EHI_180800 |
| 146 | hypothetical protein | EHI_018140 |
| 146 | hypothetical protein | EHI_062990 |
| 150 | hypothetical protein | EHI_044720 |
| 150 | hypothetical protein | EHI_197100 |
| 150 | hypothetical protein, conserved | EHI_002000 |
| 150 | hypothetical protein, conserved | EHI_087400 |
| 150 | hypothetical protein, conserved | EHI_129640 |
| 150 | hypothetical protein, conserved | EHI_162220 |
| 152 | hypothetical protein | EHI_002230 |
| 152 | hypothetical protein | EHI_018020 |
| 152 | hypothetical protein | EHI_029510 |
| 152 | hypothetical protein | EHI_037690 |
| 152 | hypothetical protein | EHI_132850 |
| 152 | hypothetical protein | EHI_196710 |
| 154 | hypothetical protein | EHI_034820 |
| 154 | hypothetical protein | EHI_072510 |
| 154 | hypothetical protein | EHI_091690 |
| 154 | hypothetical protein | EHI_119770 |
| 154 | hypothetical protein | EHI_172020 |
| 154 | hypothetical protein | EHI_173600 |
| 157 | GalNAc lectin light subunit | EHI_148790 |
| 157 | galactose-inhibitable lectin, putative | EHI_058330 |
| 157 | galactose binding lectin 35 kDa subunit, putative | EHI_027800 |
| 157 | galactose-specific adhesin light subunit, putative | EHI_049690 |
| 157 | galactose-inhibitable lectin 35 kda subunit precur | EHI_035690 |
| 157 | galactose-inhibitable lectin 35 kDa subunit precur | EHI_183400 |
| 159 | hypothetical protein | EHI_092580 |
| 159 | hypothetical protein | EHI_094150 |
| 159 | hypothetical protein | EHI_148740 |
| 159 | hypothetical protein | EHI_160970 |
| 159 | hypothetical protein | EHI_164330 |
| 159 | hypothetical protein | EHI_192430 |
| 161 | hypothetical protein | EHI_011250 |
| 161 | hypothetical protein | EHI_033890 |
| 161 | hypothetical protein | EHI_071360 |
| 161 | hypothetical protein | EHI_142680 |
| 161 | hypothetical protein | EHI_193810 |
| 161 | hypothetical protein | EHI_101410 |
| 162 | hypothetical protein | EHI_034830 |
| 162 | hypothetical protein | EHI_072500 |
| 162 | hypothetical protein | EHI_091700 |
| 162 | hypothetical protein | EHI_119760 |
| 162 | hypothetical protein | EHI_172010 |
| 162 | hypothetical protein | EHI_173610 |
| 163 | hypothetical protein | EHI_068280 |
| 163 | hypothetical protein | EHI_086090 |
| 163 | hypothetical protein | EHI_125970 |
| 163 | hypothetical protein | EHI_129780 |
| 163 | hypothetical protein | EHI_197390 |
| 163 | hypothetical membrane-spanning protein | EHI_092670 |
| 167 | hypothetical protein | EHI_032470 |
| 167 | hypothetical protein | EHI_049320 |
| 167 | hypothetical protein | EHI_077750 |
| 167 | hypothetical protein | EHI_095910 |
| 167 | hypothetical protein | EHI_170940 |
| 167 | hypothetical protein | EHI_180940 |
| 171 | hypothetical protein | EHI_004440 |
| 171 | hypothetical protein | EHI_004430 |
| 171 | hypothetical protein | EHI_012080 |
| 171 | hypothetical protein | EHI_018610 |
| 171 | hypothetical protein | EHI_068440 |
| 171 | hypothetical protein | EHI_183090 |
| 180 | hypothetical protein | EHI_034570 |
| 180 | hypothetical protein | EHI_059320 |
| 180 | hypothetical protein | EHI_070910 |
| 180 | hypothetical protein | EHI_193650 |
| 180 | hypothetical protein | EHI_116950 |
| 180 | hypothetical protein | EHI_144160 |
| 183 | hypothetical protein | EHI_046040 |
| 183 | hypothetical protein | EHI_104230 |
| 183 | hypothetical protein | EHI_110000 |
| 183 | hypothetical protein | EHI_133780 |
| 183 | hypothetical protein | EHI_004060 |
| 185 | hypothetical protein | EHI_035670 |
| 185 | hypothetical protein | EHI_108710 |
| 185 | hypothetical protein | EHI_125150 |
| 185 | hypothetical protein | EHI_139010 |
| 185 | hypothetical protein | EHI_139020 |
| 186 | hypothetical protein | EHI_020930 |
| 186 | hypothetical protein | EHI_062350 |
| 186 | hypothetical protein | EHI_095500 |
| 186 | hypothetical protein | EHI_130540 |
| 186 | hypothetical protein | EHI_154750 |
| 189 | hypothetical protein | EHI_040810 |
| 189 | hypothetical protein | EHI_049930 |
| 189 | hypothetical protein | EHI_094130 |
| 189 | hypothetical protein | EHI_140210 |
| 189 | hypothetical protein | EHI_156440 |
| 190 | membrane transporter, putative | EHI_153380 |
| 190 | hypothetical protein, conserved | EHI_019090 |
| 190 | hypothetical protein, conserved | EHI_050950 |
| 190 | hypothetical protein, conserved | EHI_109690 |
| 190 | hypothetical protein, conserved | EHI_160010 |
| 192 | hypothetical protein | EHI_002110 |
| 192 | hypothetical protein | EHI_006300 |
| 192 | hypothetical protein | EHI_115820 |
| 192 | hypothetical protein | EHI_171550 |
| 192 | hypothetical protein, conserved | EHI_004620 |
| 196 | hypothetical protein | EHI_004410 |
| 196 | hypothetical protein | EHI_005160 |
| 196 | hypothetical protein | EHI_147860 |
| 196 | hypothetical protein | EHI_187400 |
| 196 | hypothetical protein | EHI_194050 |
| 2 | hypothetical protein | EHI_098720 |
| 2 | hypothetical protein | EHI_151590 |
| 2 | hypothetical protein | EHI_154590 |
| 2 | hypothetical protein | EHI_186220 |
| 2 | hypothetical protein | EHI_102700 |
| 2 | leucine rich repeat protein 1 | EHI_033560 |
| 2 | leucine rich repeat protein 1 | EHI_124070 |
| 2 | leucine rich repeat protein, BspA family | EHI_000760 |
| 2 | leucine rich repeat protein, BspA family | EHI_002120 |
| 2 | leucine rich repeat protein, BspA family | EHI_003380 |
| 2 | leucine rich repeat protein, BspA family | EHI_004360 |
| 2 | leucine rich repeat protein, BspA family | EHI_006310 |
| 2 | leucine rich repeat protein, BspA family | EHI_010000 |
| 2 | leucine rich repeat protein, BspA family | EHI_012090 |
| 2 | leucine rich repeat protein, BspA family | EHI_015120 |
| 2 | leucine rich repeat protein, BspA family | EHI_016490 |
| 2 | leucine rich repeat protein, BspA family | EHI_017710 |
| 2 | leucine rich repeat protein, BspA family | EHI_018840 |
| 2 | leucine rich repeat protein, BspA family | EHI_020090 |
| 2 | leucine rich repeat protein, BspA family | EHI_024580 |
| 2 | leucine rich repeat protein, BspA family | EHI_027680 |
| 2 | leucine rich repeat protein, BspA family | EHI_028330 |
| 2 | leucine rich repeat protein, BspA family | EHI_034610 |
| 2 | leucine rich repeat protein, BspA family | EHI_038810 |
| 2 | leucine rich repeat protein, BspA family | EHI_041470 |
| 2 | leucine rich repeat protein, BspA family | EHI_046800 |
| 2 | leucine rich repeat protein, BspA family | EHI_047820 |
| 2 | leucine rich repeat protein, BspA family | EHI_048660 |
| 2 | leucine rich repeat protein, BspA family | EHI_049160 |
| 2 | leucine rich repeat protein, BspA family | EHI_051070 |
| 2 | leucine rich repeat protein, BspA family | EHI_051080 |
| 2 | leucine rich repeat protein, BspA family | EHI_051290 |
| 2 | leucine rich repeat protein, BspA family | EHI_054160 |
| 2 | leucine rich repeat protein, BspA family | EHI_062750 |
| 2 | leucine rich repeat protein, BspA family | EHI_063030 |
| 2 | leucine rich repeat protein, BspA family | EHI_066620 |
| 2 | leucine rich repeat protein, BspA family | EHI_069190 |
| 2 | leucine rich repeat protein, BspA family | EHI_070440 |
| 2 | leucine rich repeat protein, BspA family | EHI_070330 |
| 2 | leucine rich repeat protein, BspA family | EHI_072070 |
| 2 | leucine rich repeat protein, BspA family | EHI_075960 |
| 2 | leucine rich repeat protein, BspA family | EHI_077280 |
| 2 | leucine rich repeat protein, BspA family | EHI_078570 |
| 2 | leucine rich repeat protein, BspA family | EHI_079970 |
| 2 | leucine rich repeat protein, BspA family | EHI_082060 |
| 2 | leucine rich repeat protein, BspA family | EHI_084160 |
| 2 | leucine rich repeat protein, BspA family | EHI_088100 |
| 2 | leucine rich repeat protein, BspA family | EHI_094080 |
| 2 | leucine rich repeat protein, BspA family | EHI_095060 |
| 2 | leucine rich repeat protein, BspA family | EHI_100700 |
| 2 | leucine rich repeat protein, BspA family | EHI_102380 |
| 2 | leucine rich repeat protein, BspA family | EHI_105370 |
| 2 | leucine rich repeat protein, BspA family | EHI_107220 |
| 2 | leucine rich repeat protein, BspA family | EHI_110760 |
| 2 | leucine rich repeat protein, BspA family | EHI_110590 |
| 2 | leucine rich repeat protein, BspA family | EHI_112030 |
| 2 | leucine rich repeat protein, BspA family | EHI_111960 |
| 2 | leucine rich repeat protein, BspA family | EHI_112290 |
| 2 | leucine rich repeat protein, BspA family | EHI_112690 |
| 2 | leucine rich repeat protein, BspA family | EHI_113310 |
| 2 | leucine rich repeat protein, BspA family | EHI_113190 |
| 2 | leucine rich repeat protein, BspA family | EHI_113990 |
| 2 | leucine rich repeat protein, BspA family | EHI_119470 |
| 2 | leucine rich repeat protein, BspA family | EHI_120570 |
| 2 | leucine rich repeat protein, BspA family | EHI_122410 |
| 2 | leucine rich repeat protein, BspA family | EHI_123820 |
| 2 | leucine rich repeat protein, BspA family | EHI_127100 |
| 2 | leucine rich repeat protein, BspA family | EHI_127710 |
| 2 | leucine rich repeat protein, BspA family | EHI_128460 |
| 2 | leucine rich repeat protein, BspA family | EHI_129870 |
| 2 | leucine rich repeat protein, BspA family | EHI_131490 |
| 2 | leucine rich repeat protein, BspA family | EHI_137910 |
| 2 | leucine rich repeat protein, BspA family | EHI_139390 |
| 2 | leucine rich repeat protein, BspA family | EHI_139430 |
| 2 | leucine rich repeat protein, BspA family | EHI_147680 |
| 2 | leucine rich repeat protein, BspA family | EHI_148530 |
| 2 | leucine rich repeat protein, BspA family | EHI_148460 |
| 2 | leucine rich repeat protein, BspA family | EHI_151330 |
| 2 | leucine rich repeat protein, BspA family | EHI_152950 |
| 2 | leucine rich repeat protein, BspA family | EHI_154170 |
| 2 | leucine rich repeat protein, BspA family | EHI_166160 |
| 2 | leucine rich repeat protein, BspA family | EHI_173850 |
| 2 | leucine rich repeat protein, BspA family | EHI_182250 |
| 2 | leucine rich repeat protein, BspA family | EHI_184260 |
| 2 | leucine rich repeat protein, BspA family | EHI_189090 |
| 2 | leucine rich repeat protein, BspA family | EHI_190890 |
| 2 | leucine rich repeat protein, BspA family | EHI_191510 |
| 2 | leucine rich repeat protein, BspA family | EHI_192250 |
| 2 | leucine rich repeat protein, BspA family | EHI_192600 |
| 2 | leucine rich repeat protein, BspA family | EHI_194290 |
| 2 | leucine rich repeat protein, BspA family | EHI_198630 |
| 2 | leucine rich repeat protein, BspA family | EHI_199270 |
| 2 | leucine rich repeat protein, BspA family | EHI_003790 |
| 2 | leucine rich repeat protein, BspA family | EHI_005660 |
| 2 | leucine rich repeat protein, BspA family | EHI_013750 |
| 2 | leucine rich repeat protein, BspA family | EHI_016240 |
| 2 | leucine rich repeat protein, BspA family | EHI_042470 |
| 2 | leucine rich repeat protein, BspA family | EHI_064920 |
| 2 | leucine rich repeat protein, BspA family | EHI_070230 |
| 2 | leucine rich repeat protein, BspA family | EHI_103140 |
| 2 | leucine rich repeat protein, BspA family | EHI_106460 |
| 2 | leucine rich repeat protein, BspA family | EHI_115500 |
| 2 | leucine rich repeat protein, BspA family | EHI_134140 |
| 2 | leucine rich repeat protein, BspA family | EHI_143230 |
| 2 | leucine rich repeat protein, BspA family | EHI_150150 |
| 2 | leucine rich repeat protein, BspA family | EHI_153950 |
| 2 | leucine rich repeat protein, BspA family | EHI_158740 |
| 2 | leucine rich repeat protein, BspA family | EHI_161300 |
| 2 | leucine rich repeat protein, BspA family | EHI_163960 |
| 2 | leucine rich repeat protein, BspA family | EHI_168610 |
| 2 | leucine rich repeat protein, BspA family | EHI_176480 |
| 2 | leucine rich repeat protein, BspA family | EHI_180550 |
| 20 | hypothetical protein | EHI_051190 |
| 20 | hypothetical protein | EHI_072740 |
| 20 | hypothetical protein | EHI_076370 |
| 20 | hypothetical protein | EHI_125010 |
| 20 | hypothetical protein | EHI_128900 |
| 20 | hypothetical protein | EHI_145150 |
| 20 | hypothetical protein | EHI_003690 |
| 20 | hypothetical protein | EHI_021880 |
| 20 | hypothetical protein | EHI_047520 |
| 20 | hypothetical protein | EHI_066090 |
| 20 | hypothetical protein | EHI_085170 |
| 20 | hypothetical protein | EHI_198110 |
| 208 | surface antigen ariel1, putative | EHI_098180 |
| 208 | surface antigen ariel1, putative | EHI_131360 |
| 208 | surface antigen ariel1, putative | EHI_169800 |
| 208 | surface antigen ariel1, putative | EHI_172850 |
| 208 | surface antigen ariel1, putative | EHI_101730 |
| 209 | hypothetical protein | EHI_028540 |
| 209 | hypothetical protein | EHI_076790 |
| 209 | hypothetical protein | EHI_079310 |
| 209 | hypothetical protein | EHI_079280 |
| 209 | hypothetical protein | EHI_028670 |
| 210 | hypothetical protein | EHI_017750 |
| 210 | hypothetical protein | EHI_048240 |
| 210 | hypothetical protein | EHI_107130 |
| 210 | hypothetical protein | EHI_114140 |
| 210 | hypothetical protein | EHI_117850 |
| 213 | hypothetical protein | EHI_046030 |
| 213 | hypothetical protein | EHI_104220 |
| 213 | hypothetical protein | EHI_109990 |
| 213 | hypothetical protein | EHI_004050 |
| 213 | hypothetical protein | EHI_157670 |
| 214 | hypothetical protein | EHI_049350 |
| 214 | hypothetical protein | EHI_142960 |
| 214 | hypothetical protein | EHI_179320 |
| 214 | hypothetical protein, conserved | EHI_025100 |
| 214 | hypothetical protein, conserved | EHI_139040 |
| 216 | hypothetical protein | EHI_072480 |
| 216 | hypothetical protein | EHI_091720 |
| 216 | hypothetical protein | EHI_119740 |
| 216 | hypothetical protein | EHI_171990 |
| 216 | hypothetical protein | EHI_173630 |
| 218 | hypothetical protein, conserved | EHI_041580 |
| 218 | hypothetical protein, conserved | EHI_093890 |
| 218 | hypothetical protein, conserved | EHI_098440 |
| 218 | hypothetical protein, conserved | EHI_175120 |
| 218 | hypothetical protein, conserved | EHI_175400 |
| 220 | hypothetical protein | EHI_072460 |
| 220 | hypothetical protein | EHI_091740 |
| 220 | hypothetical protein | EHI_119720 |
| 220 | hypothetical protein | EHI_171980 |
| 220 | hypothetical protein | EHI_173650 |
| 221 | hypothetical protein | EHI_050460 |
| 221 | hypothetical protein | EHI_050390 |
| 221 | hypothetical protein | EHI_058480 |
| 221 | hypothetical protein | EHI_183410 |
| 221 | hypothetical protein | EHI_199010 |
| 228 | GalGalNAc lectin heavy subunit | EHI_012270 |
| 228 | galactose-specific adhesin 170kD subunit | EHI_077500 |
| 228 | GalGalNAc lectin heavy subunit, putative | EHI_046650 |
| 228 | galactose-specific adhesin 170kD subunit, putative | EHI_042370 |
| 228 | galactose-inhibitable lectin 170 kDa subunit, puta | EHI_133900 |
| 229 | hypothetical protein | EHI_013930 |
| 229 | hypothetical protein | EHI_028010 |
| 229 | hypothetical protein | EHI_057320 |
| 229 | hypothetical protein | EHI_105470 |
| 229 | hypothetical protein | EHI_128330 |
| 231 | hypothetical protein | EHI_070560 |
| 231 | hypothetical protein | EHI_101130 |
| 231 | hypothetical protein | EHI_127690 |
| 231 | hypothetical protein | EHI_166040 |
| 231 | hypothetical protein | EHI_007140 |
| 234 | hypothetical protein | EHI_049760 |
| 234 | hypothetical protein | EHI_181400 |
| 234 | hypothetical protein | EHI_017770 |
| 234 | hypothetical protein | EHI_017780 |
| 234 | hypothetical protein | EHI_093230 |
| 237 | hypothetical protein | EHI_002240 |
| 237 | hypothetical protein | EHI_029500 |
| 237 | hypothetical protein | EHI_037700 |
| 237 | hypothetical protein | EHI_196720 |
| 237 | hypothetical protein | EHI_018030 |
| 238 | hypothetical protein | EHI_174630 |
| 238 | hypothetical protein | EHI_174380 |
| 238 | hypothetical protein | EHI_014510 |
| 238 | hypothetical protein | EHI_038030 |
| 238 | hypothetical protein | EHI_074940 |
| 239 | hypothetical protein | EHI_121810 |
| 239 | hypothetical protein | EHI_125330 |
| 239 | hypothetical protein | EHI_161020 |
| 239 | hypothetical protein | EHI_178730 |
| 239 | hypothetical protein | EHI_198920 |
| 31 | hypothetical protein | EHI_012140 |
| 31 | hypothetical protein | EHI_019140 |
| 31 | hypothetical protein | EHI_021320 |
| 31 | hypothetical protein | EHI_049800 |
| 31 | hypothetical protein | EHI_050860 |
| 31 | hypothetical protein | EHI_060750 |
| 31 | hypothetical protein | EHI_100450 |
| 31 | hypothetical protein | EHI_110370 |
| 31 | hypothetical protein | EHI_122910 |
| 31 | hypothetical protein | EHI_137240 |
| 31 | hypothetical protein | EHI_140160 |
| 31 | hypothetical protein | EHI_142070 |
| 31 | hypothetical protein | EHI_159900 |
| 31 | hypothetical protein | EHI_183550 |
| 31 | hypothetical protein | EHI_197090 |
| 31 | hypothetical protein | EHI_201230 |
| 31 | hypothetical protein, conserved | EHI_006760 |
| 31 | hypothetical protein, conserved | EHI_012150 |
| 34 | hypothetical protein | EHI_006740 |
| 34 | hypothetical protein | EHI_027790 |
| 34 | hypothetical protein | EHI_064510 |
| 34 | hypothetical protein | EHI_072090 |
| 34 | hypothetical protein | EHI_083660 |
| 34 | hypothetical protein | EHI_105190 |
| 34 | hypothetical protein | EHI_105260 |
| 34 | hypothetical protein | EHI_105320 |
| 34 | hypothetical protein | EHI_112040 |
| 34 | hypothetical protein | EHI_122890 |
| 34 | hypothetical protein | EHI_142010 |
| 34 | hypothetical protein | EHI_149030 |
| 34 | hypothetical protein | EHI_153210 |
| 34 | hypothetical protein | EHI_156460 |
| 34 | hypothetical protein | EHI_178450 |
| 34 | hypothetical protein | EHI_187050 |
| 34 | hypothetical protein | EHI_194560 |
| 36 | hypothetical protein | EHI_012440 |
| 36 | hypothetical protein | EHI_019130 |
| 36 | hypothetical protein | EHI_049870 |
| 36 | hypothetical protein | EHI_057690 |
| 36 | hypothetical protein | EHI_062470 |
| 36 | hypothetical protein | EHI_065260 |
| 36 | hypothetical protein | EHI_079870 |
| 36 | hypothetical protein | EHI_106070 |
| 36 | hypothetical protein | EHI_110480 |
| 36 | hypothetical protein | EHI_125400 |
| 36 | hypothetical protein | EHI_128470 |
| 36 | hypothetical protein | EHI_163590 |
| 36 | hypothetical protein | EHI_166360 |
| 36 | hypothetical protein | EHI_059900 |
| 36 | hypothetical protein | EHI_095710 |
| 42 | hypothetical protein, conserved | EHI_011270 |
| 42 | hypothetical protein, conserved | EHI_018390 |
| 42 | hypothetical protein, conserved | EHI_033550 |
| 42 | hypothetical protein, conserved | EHI_051400 |
| 42 | hypothetical protein, conserved | EHI_057420 |
| 42 | hypothetical protein, conserved | EHI_073060 |
| 42 | hypothetical protein, conserved | EHI_077290 |
| 42 | hypothetical protein, conserved | EHI_090940 |
| 42 | hypothetical protein, conserved | EHI_107540 |
| 42 | hypothetical protein, conserved | EHI_114950 |
| 42 | hypothetical protein, conserved | EHI_144490 |
| 42 | hypothetical protein, conserved | EHI_181810 |
| 42 | hypothetical protein, conserved | EHI_037340 |
| 42 | hypothetical protein, conserved | EHI_101630 |
| 43 | hypothetical protein | EHI_034530 |
| 43 | hypothetical protein | EHI_045820 |
| 43 | hypothetical protein | EHI_059280 |
| 43 | hypothetical protein | EHI_074520 |
| 43 | hypothetical protein | EHI_154550 |
| 43 | hypothetical protein | EHI_162780 |
| 43 | hypothetical protein | EHI_193690 |
| 43 | hypothetical protein | EHI_018270 |
| 43 | hypothetical protein | EHI_020830 |
| 43 | hypothetical protein | EHI_028770 |
| 43 | hypothetical protein | EHI_136840 |
| 43 | hypothetical protein | EHI_196760 |
| 48 | hypothetical protein, conserved | EHI_011260 |
| 48 | hypothetical protein, conserved | EHI_033910 |
| 48 | hypothetical protein, conserved | EHI_104900 |
| 48 | hypothetical protein, conserved | EHI_104890 |
| 48 | hypothetical protein, conserved | EHI_142670 |
| 48 | hypothetical protein, conserved | EHI_177040 |
| 48 | hypothetical protein, conserved | EHI_193790 |
| 48 | hypothetical protein, conserved | EHI_193800 |
| 48 | hypothetical protein, conserved | EHI_101420 |
| 48 | hypothetical protein, conserved | EHI_101430 |
| 49 | hypothetical protein | EHI_027020 |
| 49 | hypothetical protein | EHI_034510 |
| 49 | hypothetical protein | EHI_059260 |
| 49 | hypothetical protein | EHI_074540 |
| 49 | hypothetical protein | EHI_078530 |
| 49 | hypothetical protein | EHI_154570 |
| 49 | hypothetical protein | EHI_162800 |
| 49 | hypothetical protein | EHI_018290 |
| 49 | hypothetical protein | EHI_029940 |
| 49 | hypothetical protein | EHI_056810 |
| 49 | hypothetical protein | EHI_142570 |
| 49 | hypothetical protein | EHI_165670 |
| 49 | hypothetical protein | EHI_196780 |
| 49 | hypothetical protein | EHI_200440 |
| 5 | hypothetical protein, conserved | EHI_004530 |
| 5 | hypothetical protein, conserved | EHI_007020 |
| 5 | hypothetical protein, conserved | EHI_006950 |
| 5 | hypothetical protein, conserved | EHI_008020 |
| 5 | hypothetical protein, conserved | EHI_008610 |
| 5 | hypothetical protein, conserved | EHI_010300 |
| 5 | hypothetical protein, conserved | EHI_014150 |
| 5 | hypothetical protein, conserved | EHI_016350 |
| 5 | hypothetical protein, conserved | EHI_022130 |
| 5 | hypothetical protein, conserved | EHI_023490 |
| 5 | hypothetical protein, conserved | EHI_025370 |
| 5 | hypothetical protein, conserved | EHI_030150 |
| 5 | hypothetical protein, conserved | EHI_033690 |
| 5 | hypothetical protein, conserved | EHI_035820 |
| 5 | hypothetical protein, conserved | EHI_039330 |
| 5 | hypothetical protein, conserved | EHI_047210 |
| 5 | hypothetical protein, conserved | EHI_053160 |
| 5 | hypothetical protein, conserved | EHI_054520 |
| 5 | hypothetical protein, conserved | EHI_055390 |
| 5 | hypothetical protein, conserved | EHI_059690 |
| 5 | hypothetical protein, conserved | EHI_066720 |
| 5 | hypothetical protein, conserved | EHI_076270 |
| 5 | hypothetical protein, conserved | EHI_076250 |
| 5 | hypothetical protein, conserved | EHI_079960 |
| 5 | hypothetical protein, conserved | EHI_080710 |
| 5 | hypothetical protein, conserved | EHI_087350 |
| 5 | hypothetical protein, conserved | EHI_092080 |
| 5 | hypothetical protein, conserved | EHI_092610 |
| 5 | hypothetical protein, conserved | EHI_098260 |
| 5 | hypothetical protein, conserved | EHI_099670 |
| 5 | hypothetical protein, conserved | EHI_110960 |
| 5 | hypothetical protein, conserved | EHI_111550 |
| 5 | hypothetical protein, conserved | EHI_113950 |
| 5 | hypothetical protein, conserved | EHI_121060 |
| 5 | hypothetical protein, conserved | EHI_122900 |
| 5 | hypothetical protein, conserved | EHI_123250 |
| 5 | hypothetical protein, conserved | EHI_131140 |
| 5 | hypothetical protein, conserved | EHI_131480 |
| 5 | hypothetical protein, conserved | EHI_133940 |
| 5 | hypothetical protein, conserved | EHI_133530 |
| 5 | hypothetical protein, conserved | EHI_134780 |
| 5 | hypothetical protein, conserved | EHI_140300 |
| 5 | hypothetical protein, conserved | EHI_140150 |
| 5 | hypothetical protein, conserved | EHI_143550 |
| 5 | hypothetical protein, conserved | EHI_143620 |
| 5 | hypothetical protein, conserved | EHI_152160 |
| 5 | hypothetical protein, conserved | EHI_153300 |
| 5 | hypothetical protein, conserved | EHI_152870 |
| 5 | hypothetical protein, conserved | EHI_156340 |
| 5 | hypothetical protein, conserved | EHI_174160 |
| 5 | hypothetical protein, conserved | EHI_176850 |
| 5 | hypothetical protein, conserved | EHI_177190 |
| 5 | hypothetical protein, conserved | EHI_182510 |
| 5 | hypothetical protein, conserved | EHI_192420 |
| 5 | hypothetical protein, conserved | EHI_194870 |
| 5 | hypothetical protein, conserved | EHI_199170 |
| 5 | hypothetical protein, conserved | EHI_199130 |
| 5 | hypothetical protein, conserved | EHI_034010 |
| 5 | hypothetical protein, conserved | EHI_183880 |
| 5 | hypothetical protein, conserved | EHI_195970 |
| 5 | kinetochore protein Spc25 domain-containing protei | EHI_181520 |
| 52 | hypothetical protein | EHI_109260 |
| 52 | hypothetical protein | EHI_181910 |
| 54 | hypothetical protein | EHI_005030 |
| 54 | hypothetical protein | EHI_006040 |
| 54 | hypothetical protein | EHI_016370 |
| 54 | hypothetical protein | EHI_017660 |
| 54 | hypothetical protein | EHI_021550 |
| 54 | hypothetical protein | EHI_027500 |
| 54 | hypothetical protein | EHI_108690 |
| 54 | hypothetical protein | EHI_129880 |
| 54 | hypothetical protein | EHI_134600 |
| 54 | hypothetical protein | EHI_150450 |
| 54 | hypothetical protein | EHI_150420 |
| 54 | hypothetical protein | EHI_154310 |
| 54 | hypothetical protein | EHI_039960 |
| 56 | hypothetical protein | EHI_001740 |
| 56 | hypothetical protein | EHI_174620 |
| 56 | hypothetical protein | EHI_059150 |
| 56 | hypothetical protein | EHI_074930 |
| 56 | hypothetical protein | EHI_125500 |
| 58 | hypothetical protein | EHI_027010 |
| 58 | hypothetical protein | EHI_034520 |
| 58 | hypothetical protein | EHI_059270 |
| 58 | hypothetical protein | EHI_078520 |
| 58 | hypothetical protein | EHI_014610 |
| 58 | hypothetical protein | EHI_056800 |
| 58 | hypothetical protein | EHI_165660 |
| 58 | hypothetical protein | EHI_196770 |
| 58 | hypothetical protein | EHI_200450 |
| 64 | hypothetical protein | EHI_001730 |
| 64 | hypothetical protein | EHI_109240 |
| 64 | hypothetical protein | EHI_168710 |
| 64 | hypothetical protein | EHI_174610 |
| 64 | hypothetical protein | EHI_020900 |
| 64 | hypothetical protein | EHI_041170 |
| 64 | hypothetical protein | EHI_042840 |
| 64 | hypothetical protein | EHI_059160 |
| 64 | hypothetical protein | EHI_113790 |
| 64 | hypothetical protein | EHI_149130 |
| 66 | hypothetical protein | EHI_047000 |
| 66 | hypothetical protein | EHI_108990 |
| 66 | hypothetical protein | EHI_104000 |
| 66 | hypothetical protein | EHI_195770 |
| 67 | hypothetical protein | EHI_019630 |
| 67 | hypothetical protein | EHI_046050 |
| 67 | hypothetical protein | EHI_052410 |
| 67 | hypothetical protein | EHI_062960 |
| 67 | hypothetical protein | EHI_067090 |
| 67 | hypothetical protein | EHI_077530 |
| 67 | hypothetical protein | EHI_093990 |
| 67 | hypothetical protein | EHI_146120 |
| 67 | hypothetical protein | EHI_191400 |
| 67 | hypothetical protein | EHI_004070 |
| 67 | hypothetical protein | EHI_195870 |
| 70 | hypothetical protein | EHI_034550 |
| 70 | hypothetical protein | EHI_059300 |
| 70 | hypothetical protein | EHI_074510 |
| 70 | hypothetical protein | EHI_162770 |
| 70 | hypothetical protein | EHI_190310 |
| 70 | hypothetical protein | EHI_193670 |
| 70 | hypothetical protein | EHI_018150 |
| 70 | hypothetical protein | EHI_020710 |
| 70 | hypothetical protein | EHI_072950 |
| 70 | hypothetical protein | EHI_116930 |
| 70 | hypothetical protein | EHI_196740 |
| 72 | hypothetical protein | EHI_011240 |
| 72 | hypothetical protein | EHI_033870 |
| 72 | hypothetical protein | EHI_071340 |
| 72 | hypothetical protein | EHI_104920 |
| 72 | hypothetical protein | EHI_118830 |
| 72 | hypothetical protein | EHI_142690 |
| 72 | hypothetical protein | EHI_177020 |
| 72 | hypothetical protein | EHI_178760 |
| 72 | hypothetical protein | EHI_193820 |
| 72 | hypothetical protein | EHI_101400 |
| 76 | hypothetical protein | EHI_020980 |
| 76 | hypothetical protein | EHI_062300 |
| 76 | hypothetical protein | EHI_072520 |
| 76 | hypothetical protein | EHI_089450 |
| 76 | hypothetical protein | EHI_091680 |
| 76 | hypothetical protein | EHI_095310 |
| 76 | hypothetical protein | EHI_095450 |
| 76 | hypothetical protein | EHI_119790 |
| 76 | hypothetical protein | EHI_130490 |
| 76 | hypothetical protein | EHI_154700 |
| 8 | hypothetical protein | EHI_012550 |
| 8 | hypothetical protein | EHI_018950 |
| 8 | hypothetical protein | EHI_031950 |
| 8 | hypothetical protein | EHI_047620 |
| 8 | hypothetical protein | EHI_067200 |
| 8 | hypothetical protein | EHI_067600 |
| 8 | hypothetical protein | EHI_070790 |
| 8 | hypothetical protein | EHI_076380 |
| 8 | hypothetical protein | EHI_077760 |
| 8 | hypothetical protein | EHI_109250 |
| 8 | hypothetical protein | EHI_118070 |
| 8 | hypothetical protein | EHI_120590 |
| 8 | hypothetical protein | EHI_137370 |
| 8 | hypothetical protein | EHI_150500 |
| 8 | hypothetical protein | EHI_151740 |
| 8 | hypothetical protein | EHI_160450 |
| 8 | hypothetical protein | EHI_170960 |
| 8 | hypothetical protein | EHI_172620 |
| 8 | hypothetical protein | EHI_179700 |
| 8 | hypothetical protein | EHI_180400 |
| 8 | hypothetical protein | EHI_185260 |
| 8 | hypothetical protein | EHI_013440 |
| 8 | hypothetical protein | EHI_047510 |
| 8 | hypothetical protein | EHI_047630 |
| 8 | hypothetical protein | EHI_061140 |
| 8 | hypothetical protein | EHI_068850 |
| 8 | hypothetical protein | EHI_069830 |
| 8 | hypothetical protein | EHI_085160 |
| 8 | hypothetical protein | EHI_089000 |
| 8 | hypothetical protein | EHI_089570 |
| 8 | hypothetical protein | EHI_094580 |
| 8 | hypothetical protein | EHI_094570 |
| 8 | hypothetical protein | EHI_122200 |
| 8 | hypothetical protein | EHI_129100 |
| 8 | hypothetical protein | EHI_139190 |
| 8 | hypothetical protein | EHI_145160 |
| 8 | hypothetical protein | EHI_157460 |
| 8 | hypothetical protein | EHI_162910 |
| 81 | hypothetical protein | EHI_020960 |
| 81 | hypothetical protein | EHI_062320 |
| 81 | hypothetical protein | EHI_074570 |
| 81 | hypothetical protein | EHI_089430 |
| 81 | hypothetical protein | EHI_130510 |
| 81 | hypothetical protein | EHI_145300 |
| 81 | hypothetical protein | EHI_154720 |
| 83 | hypothetical protein | EHI_090430 |
| 83 | hypothetical protein | EHI_092490 |
| 83 | hypothetical protein | EHI_114190 |
| 83 | hypothetical protein | EHI_140740 |
| 83 | hypothetical protein | EHI_031640 |
| 83 | sulfotransferase, putative | EHI_197340 |
| 83 | hypothetical protein, conserved | EHI_146990 |
| 83 | hypothetical protein, conserved | EHI_166030 |
| 83 | hypothetical protein, conserved | EHI_181190 |
| 85 | hypothetical protein | EHI_020920 |
| 85 | hypothetical protein | EHI_062360 |
| 85 | hypothetical protein | EHI_095510 |
| 85 | hypothetical protein | EHI_119750 |
| 85 | hypothetical protein | EHI_130550 |
| 85 | hypothetical protein | EHI_145340 |
| 85 | hypothetical protein | EHI_154760 |
| 93 | hypothetical protein | EHI_003270 |
| 93 | hypothetical protein | EHI_003580 |
| 93 | hypothetical protein | EHI_005090 |
| 93 | hypothetical protein | EHI_012700 |
| 93 | hypothetical protein | EHI_045040 |
| 93 | hypothetical protein | EHI_104200 |
| 93 | hypothetical protein | EHI_106360 |
| 93 | hypothetical protein | EHI_128490 |
| 93 | hypothetical protein | EHI_131460 |
| 95 | hypothetical protein | EHI_014280 |
| 95 | hypothetical protein | EHI_050730 |
| 95 | hypothetical protein | EHI_056670 |
| 95 | hypothetical protein | EHI_129900 |
| 95 | hypothetical protein | EHI_151970 |
| 95 | hypothetical protein | EHI_159470 |
| 95 | hypothetical protein | EHI_178790 |
| 95 | hypothetical protein | EHI_178800 |
| 96 | hypothetical protein | EHI_009920 |
| 96 | hypothetical protein | EHI_009900 |
| 96 | hypothetical protein | EHI_050960 |
| 96 | hypothetical protein | EHI_103570 |
| 96 | hypothetical protein | EHI_103890 |
| 96 | hypothetical protein | EHI_127150 |
| 96 | hypothetical protein | EHI_142930 |
| 96 | hypothetical protein | EHI_201240 |
| 97 | hypothetical protein | EHI_010350 |
| 97 | hypothetical protein | EHI_049060 |
| 97 | hypothetical protein | EHI_054490 |
| 97 | hypothetical protein | EHI_054480 |
| 97 | hypothetical protein | EHI_096410 |
| 97 | hypothetical protein | EHI_170110 |
| 97 | hypothetical protein | EHI_183470 |
| 97 | hypothetical protein | EHI_129370 |
| 99 | hypothetical protein | EHI_000650 |
| 99 | hypothetical protein | EHI_001920 |
| 99 | hypothetical protein | EHI_015990 |
| 99 | hypothetical protein | EHI_037200 |
| 99 | hypothetical protein | EHI_054200 |
| 99 | hypothetical protein | EHI_062550 |
| 99 | hypothetical protein, conserved | EHI_068100 |
| 99 | hypothetical protein, conserved | EHI_079840 |
