# Supplementary material for: New Assembly, Reannotation and Analysis of the Entamoeba histolytica Genome Reveal New Genomic Features and Protein Content Information
Source: PLoS Negl Trop Dis. 2010 Jun 15;4(6):e716. doi: 10.1371/journal.pntd.0000716 (PMC2886108; doi:10.1371/journal.pntd.0000716)
Supplement: Table S3 — Entamoeba histolytica genes associated to Repetitive elements. Table S3 provides the complete list of E. histolytica protein families that show a close association (within 1 kb upstream or downstream) with transposable elements. (0.54 MB DOC) [file pntd.0000716.s005.doc]

| Table S3: *E. histolytica* genes associated to Repetitive elements | | |
| --- | --- | --- |
| Family ID | Protein name | Public Locus (GenBank) |
| 1 | Rab family GTPase | EHI_005460 |
| 1 | RAS-related protein racG, putative | EHI_031740 |
| 1 | Rho family GTPase | EHI_046630 |
| 1 | GTP-binding protein, putative | EHI_051090 |
| 1 | Rab family GTPase | EHI_053150 |
| 1 | small GTPase RhoA, putative | EHI_053210 |
| 1 | Rab family GTPase | EHI_056100 |
| 1 | Ras family GTPase | EHI_074750 |
| 1 | Ras family GTPase | EHI_093760 |
| 1 | Rab family GTPase | EHI_097650 |
| 1 | Rab family GTPase | EHI_114210 |
| 1 | Rab family GTPase | EHI_118920 |
| 1 | Rab family GTPase | EHI_140770 |
| 1 | Rab family GTPase | EHI_151610 |
| 1 | Rab family GTPase | EHI_168600 |
| 1 | small GTP binding protein Rab7, putative | EHI_189100 |
| 2 | leucine rich repeat protein, BspA family | EHI_003790 |
| 2 | leucine rich repeat protein, BspA family | EHI_005660 |
| 2 | leucine rich repeat protein, BspA family | EHI_016490 |
| 2 | leucine rich repeat protein, BspA family | EHI_041470 |
| 2 | leucine rich repeat protein, BspA family | EHI_042470 |
| 2 | leucine rich repeat protein, BspA family | EHI_046800 |
| 2 | leucine rich repeat protein, BspA family | EHI_049160 |
| 2 | leucine rich repeat protein, BspA family | EHI_051290 |
| 2 | leucine rich repeat protein, BspA family | EHI_066620 |
| 2 | leucine rich repeat protein, BspA family | EHI_069190 |
| 2 | leucine rich repeat protein, BspA family | EHI_070230 |
| 2 | leucine rich repeat protein, BspA family | EHI_084160 |
| 2 | leucine rich repeat protein, BspA family | EHI_102380 |
| 2 | hypothetical protein | EHI_102700 |
| 2 | leucine rich repeat protein, BspA family | EHI_103140 |
| 2 | leucine rich repeat protein, BspA family | EHI_106460 |
| 2 | leucine rich repeat protein, BspA family | EHI_111950 |
| 2 | leucine rich repeat protein, BspA family | EHI_112690 |
| 2 | leucine rich repeat protein, BspA family | EHI_113310 |
| 2 | leucine rich repeat protein, BspA family | EHI_113190 |
| 2 | leucine rich repeat protein, BspA family | EHI_115500 |
| 2 | leucine rich repeat protein, BspA family | EHI_120570 |
| 2 | leucine rich repeat protein, BspA family | EHI_122410 |
| 2 | leucine rich repeat protein, BspA family | EHI_123820 |
| 2 | leucine rich repeat protein, BspA family | EHI_128460 |
| 2 | leucine rich repeat protein, BspA family | EHI_129870 |
| 2 | leucine rich repeat protein, BspA family | EHI_134140 |
| 2 | leucine rich repeat protein, BspA family | EHI_143230 |
| 2 | leucine rich repeat protein, BspA family | EHI_151330 |
| 2 | leucine rich repeat protein, BspA family | EHI_161300 |
| 2 | leucine rich repeat protein, BspA family | EHI_163960 |
| 2 | leucine rich repeat protein, BspA family | EHI_166160 |
| 2 | leucine rich repeat protein, BspA family | EHI_168610 |
| 2 | leucine rich repeat protein, BspA family | EHI_180550 |
| 2 | leucine rich repeat protein, BspA family | EHI_182250 |
| 2 | leucine rich repeat protein, BspA family | EHI_184260 |
| 2 | hypothetical protein | EHI_186220 |
| 2 | leucine rich repeat protein, BspA family | EHI_189090 |
| 2 | leucine rich repeat protein, BspA family | EHI_190890 |
| 2 | leucine rich repeat protein, BspA family | EHI_191510 |
| 2 | leucine rich repeat protein, BspA family | EHI_199270 |
| 3 | protein kinase domain containing protein, pseudogene | EHI_022000 |
| 3 | protein kinase domain containing protein | EHI_041920 |
| 3 | protein kinase domain containing protein | EHI_069520 |
| 3 | protein kinase, putative | EHI_092530 |
| 3 | protein kinase domain containing protein, pseudogene | EHI_105810 |
| 3 | protein kinase domain containing protein | EHI_138550 |
| 3 | protein kinase domain containing protein | EHI_142710 |
| 3 | protein kinase domain containing protein | EHI_144590 |
| 3 | protein kinase , putative | EHI_193840 |
| 4 | hypothetical protein | EHI_000240 |
| 5 | hypothetical protein, conserved | EHI_004530 |
| 5 | hypothetical protein, conserved | EHI_010300 |
| 5 | hypothetical protein, conserved | EHI_035820 |
| 5 | hypothetical protein, conserved | EHI_039330 |
| 5 | hypothetical protein, conserved | EHI_047210 |
| 5 | hypothetical protein, conserved | EHI_053160 |
| 5 | hypothetical protein, conserved | EHI_099670 |
| 5 | hypothetical protein, conserved | EHI_111550 |
| 5 | hypothetical protein, conserved | EHI_121060 |
| 5 | kinetochore protein Spc25 domain-containing protein | EHI_181520 |
| 5 | hypothetical protein, conserved | EHI_194870 |
| 5 | hypothetical protein, conserved | EHI_195970 |
| 5 | hypothetical protein, conserved | EHI_199170 |
| 6 | RNA recognition motif domain containing protein | EHI_000260 |
| 6 | hypothetical protein, conserved | EHI_049380 |
| 6 | RNA recognition motif domain containing protein | EHI_067210 |
| 6 | RNA recognition motif domain containing protein | EHI_079880 |
| 7 | RhoGAP domain containing protein | EHI_094820 |
| 7 | RhoGAP domain containing protein | EHI_110800 |
| 7 | RhoGAP domain containing protein | EHI_117610 |
| 7 | RhoGAP domain containing protein | EHI_171170 |
| 8 | hypothetical protein | EHI_018950 |
| 8 | hypothetical protein | EHI_031950 |
| 8 | hypothetical protein | EHI_047620 |
| 8 | hypothetical protein | EHI_109250 |
| 8 | hypothetical protein | EHI_137370 |
| 8 | hypothetical protein | EHI_160450 |
| 8 | hypothetical protein | EHI_179700 |
| 8 | hypothetical protein | EHI_180400 |
| 8 | hypothetical protein | EHI_185260 |
| 9 | tyrosine kinase, putative | EHI_030420 |
| 9 | tyrosine kinase, putative | EHI_058930 |
| 9 | tyrosine kinase, putative | EHI_092260 |
| 9 | tyrosine kinase, putative | EHI_117680 |
| 9 | tyrosine kinase, putative | EHI_124500 |
| 9 | tyrosine kinase, putative | EHI_167650 |
| 9 | tyrosine kinase, putative | EHI_184810 |
| 9 | tyrosine kinase, putative | EHI_195050 |
| 9 | protein kinase domain containing protein | EHI_201080 |
| 10 | hypothetical protein | EHI_011400 |
| 10 | hypothetical protein | EHI_029390 |
| 10 | hypothetical protein | EHI_069090 |
| 11 | hypothetical protein | EHI_039720 |
| 11 | hypothetical protein | EHI_126200 |
| 12 | heat shock protein 70, putative | EHI_002560 |
| 12 | heat shock protein 70, putative | EHI_021780 |
| 12 | heat shock protein 70, putative | EHI_065320 |
| 12 | heat shock protein 70, putative | EHI_065770 |
| 12 | heat shock protein 70, putative | EHI_123490 |
| 12 | heat shock protein 70, putative | EHI_130160 |
| 12 | heat shock protein 70, putative | EHI_137670 |
| 12 | heat shock protein 70, putative | EHI_150770 |
| 12 | heat shock protein 70, putative | EHI_180380 |
| 12 | heat shock protein 70, putative | EHI_185120 |
| 12 | heat shock protein 70, putative | EHI_198320 |
| 13 | hypothetical protein, conserved | EHI_029600 |
| 13 | hypothetical protein, conserved | EHI_065760 |
| 13 | leucine-rich repeat containing protein | EHI_073680 |
| 13 | leucine-rich repeat containing protein | EHI_105230 |
| 13 | leucine-rich repeat containing protein | EHI_109830 |
| 14 | calmodulin-dependent calcineurin A subunit,gamma isoform, putative | EHI_115710 |
| 14 | serine/threonine protein phosphatase, putative | EHI_117570 |
| 14 | ser/thr protein phosphatase family protein | EHI_121790 |
| 14 | ser/thr protein phosphatase family protein | EHI_139350 |
| 15 | surface antigen ariel1, putative | EHI_005260 |
| 15 | hypothetical protein | EHI_015220 |
| 15 | hypothetical protein | EHI_032060 |
| 15 | dentin sialophospho protein precursor, putative | EHI_037010 |
| 15 | hypothetical protein | EHI_037920 |
| 15 | cylicin-2, putative | EHI_040060 |
| 15 | hypothetical protein | EHI_063240 |
| 15 | hypothetical protein | EHI_071210 |
| 15 | serine-rich 25 kDa antigen protein, putative | EHI_072000 |
| 15 | nucleosome-binding protein 1, putative | EHI_103900 |
| 15 | dentin sialophospho protein precursor, putative | EHI_188600 |
| 15 | hypothetical protein | EHI_200950 |
| 16 | CXXC-rich protein | EHI_050970 |
| 16 | hypothetical protein | EHI_125020 |
| 16 | Gal/GalNAc lectin Igl2, putative | EHI_183000 |
| 17 | EF-hand calcium-binding domain containing protein | EHI_060740 |
| 17 | calmodulin, putative | EHI_117470 |
| 17 | calcium binding family protein | EHI_177620 |
| 18 | guanine nucleotide exchange factor, putative | EHI_192390 |
| 19 | zinc finger domain containing protein | EHI_029000 |
| 19 | zinc finger domain containing protein | EHI_036640 |
| 19 | zinc finger domain containing protein | EHI_038330 |
| 19 | zinc finger domain containing protein | EHI_073180 |
| 19 | zinc finger domain containing protein | EHI_098040 |
| 19 | zinc finger domain containing protein | EHI_154180 |
| 21 | hypothetical protein, conserved domain containing | EHI_008430 |
| 22 | HEAT repeat domain containing protein | EHI_064450 |
| 22 | HEAT repeat domain containing protein | EHI_110640 |
| 22 | HEAT repeat domain containing protein | EHI_124530 |
| 24 | serine/threonine protein kinase, putative | EHI_048410 |
| 24 | tyrosin kinase, putative | EHI_065500 |
| 24 | tyrosin kinase, putative | EHI_097640 |
| 24 | serine/threonine protein kinase, putative | EHI_162340 |
| 25 | protein phosphatase domain-containing protein | EHI_064720 |
| 25 | protein phosphatase domain-containing protein | EHI_075640 |
| 26 | ubiquitin carboxyl-terminal hydrolase domain containing protein | EHI_043740 |
| 26 | ubiquitin carboxyl-terminal hydrolase domain containing protein | EHI_049540 |
| 26 | ubiquitin carboxyl-terminal hydrolase domain containing protein | EHI_059810 |
| 27 | myb-like DNA-binding domain containing protein | EHI_007310 |
| 27 | myb-like DNA-binding domain containing protein | EHI_098070 |
| 27 | myb-like DNA-binding domain containing protein | EHI_130060 |
| 28 | mitochondrial-type heat shock protein 70 | EHI_007150 |
| 29 | AIG1 family protein, putative | EHI_025990 |
| 29 | AIG1 family protein, putative | EHI_026000 |
| 29 | AIG1 family protein, putative | EHI_079610 |
| 29 | AIG1 family protein, putative | EHI_109120 |
| 29 | AIG1 family protein, putative | EHI_119040 |
| 29 | AIG1 family protein, putative | EHI_126560 |
| 29 | AIG1 family protein, putative | EHI_136950 |
| 29 | AIG1 family protein, putative | EHI_157260 |
| 29 | AIG1 family protein, putative | EHI_157360 |
| 29 | AIG1 family protein, putative | EHI_176580 |
| 29 | AIG1 family protein, putative | EHI_176590 |
| 29 | AIG1 family protein, putative | EHI_176700 |
| 29 | AIG1 family protein, putative | EHI_180390 |
| 29 | AIG1 family protein, putative | EHI_195260 |
| 30 | GTP-binding protein, Ras family | EHI_055420 |
| 30 | Ras family protein | EHI_094810 |
| 31 | hypothetical protein | EHI_122910 |
| 32 | DEAD/DEAH box helicase, putative | EHI_027760 |
| 32 | DEAD/DEAH box helicase, putative | EHI_052790 |
| 32 | DEAD/DEAH box helicase, putative | EHI_082600 |
| 32 | DEAD/DEAH box helicase, putative | EHI_106300 |
| 32 | DEAD/DEAH box helicase, putative | EHI_119620 |
| 33 | proteasome subunit alpha type 4, putative | EHI_167720 |
| 33 | proteasome subunit beta type 1, putative | EHI_174670 |
| 34 | hypothetical protein | EHI_083660 |
| 34 | hypothetical protein | EHI_178450 |
| 35 | Ras guanine nucleotide exchange factor, putative | EHI_127040 |
| 36 | hypothetical protein | EHI_059900 |
| 36 | hypothetical protein | EHI_125400 |
| 36 | hypothetical protein | EHI_166360 |
| 37 | hypothetical protein | EHI_067880 |
| 38 | LIM zinc finger domain containing protein | EHI_001070 |
| 38 | LIM zinc finger domain containing protein | EHI_004170 |
| 39 | transporter, major facilitator family | EHI_173950 |
| 40 | acetyltransferase, GNAT family | EHI_133980 |
| 41 | cysteine protease, putative | EHI_097900 |
| 41 | cysteine protease, putative | EHI_126170 |
| 41 | cysteine proteinase, pseudogene | EHI_144040 |
| 41 | cysteine protease, putative | EHI_180170 |
| 42 | hypothetical protein, conserved | EHI_018390 |
| 42 | hypothetical protein, conserved | EHI_033550 |
| 42 | hypothetical protein, conserved | EHI_051400 |
| 42 | hypothetical protein, conserved | EHI_057420 |
| 42 | hypothetical protein, conserved | EHI_073060 |
| 42 | hypothetical protein, conserved | EHI_077290 |
| 42 | hypothetical protein, conserved | EHI_090940 |
| 42 | hypothetical protein, conserved | EHI_101630 |
| 42 | hypothetical protein, conserved | EHI_107540 |
| 42 | hypothetical protein, conserved | EHI_114950 |
| 42 | hypothetical protein, conserved | EHI_144490 |
| 44 | ubiquitin-conjugating enzyme family protein | EHI_160740 |
| 44 | ubiquitin-conjugating enzyme family protein | EHI_178500 |
| 45 | actin, putative | EHI_163750 |
| 45 | actin, putative | EHI_163580 |
| 46 | hypothetical protein | EHI_167940 |
| 46 | hypothetical protein | EHI_188140 |
| 50 | TBC domain containing protein | EHI_010540 |
| 50 | TBC domain containing protein | EHI_110550 |
| 50 | TBC domain containing protein | EHI_166020 |
| 52 | hypothetical protein | EHI_034410 |
| 52 | hypothetical protein | EHI_036390 |
| 52 | hypothetical protein | EHI_047010 |
| 52 | hypothetical protein | EHI_109260 |
| 54 | hypothetical protein | EHI_005030 |
| 54 | hypothetical protein | EHI_016370 |
| 54 | hypothetical protein | EHI_017660 |
| 54 | hypothetical protein | EHI_134600 |
| 55 | LSM domain containing protein | EHI_025840 |
| 56 | hypothetical protein | EHI_001740 |
| 56 | hypothetical protein | EHI_020910 |
| 56 | hypothetical protein | EHI_038020 |
| 56 | hypothetical protein | EHI_042850 |
| 56 | hypothetical protein | EHI_059150 |
| 56 | hypothetical protein | EHI_074930 |
| 56 | hypothetical protein | EHI_078950 |
| 56 | hypothetical protein | EHI_079510 |
| 56 | hypothetical protein | EHI_109230 |
| 56 | hypothetical protein | EHI_125500 |
| 56 | hypothetical protein | EHI_168720 |
| 56 | hypothetical protein | EHI_174620 |
| 57 | ankyrin repeat protein, putative | EHI_139340 |
| 59 | protein tyrosine kinase domain-containing protein | EHI_128430 |
| 59 | protein tyrosine kinase domain-containing protein | EHI_148550 |
| 62 | Rap/Ran GTPase-activating protein, putative | EHI_030900 |
| 62 | Rap/Ran GTPase-activating protein, putative | EHI_090440 |
| 63 | peroxiredoxin, putative | EHI_018740 |
| 63 | peroxiredoxin | EHI_123390 |
| 63 | peroxiredoxin, putative | EHI_183180 |
| 63 | peroxiredoxin | EHI_201250 |
| 64 | hypothetical protein | EHI_001730 |
| 64 | hypothetical protein | EHI_020900 |
| 64 | hypothetical protein | EHI_041170 |
| 64 | hypothetical protein | EHI_042840 |
| 64 | hypothetical protein | EHI_059160 |
| 64 | hypothetical protein | EHI_078960 |
| 64 | hypothetical protein | EHI_109240 |
| 64 | hypothetical protein | EHI_113790 |
| 64 | hypothetical protein | EHI_149130 |
| 64 | hypothetical protein | EHI_168710 |
| 65 | endonuclease/exonuclease/phosphatase family protein | EHI_193190 |
| 66 | hypothetical protein | EHI_047000 |
| 66 | hypothetical protein | EHI_104000 |
| 66 | hypothetical protein | EHI_108990 |
| 66 | hypothetical protein | EHI_108880 |
| 66 | hypothetical protein | EHI_143220 |
| 66 | hypothetical protein | EHI_201400 |
| 67 | hypothetical protein | EHI_052410 |
| 67 | hypothetical protein | EHI_077530 |
| 67 | hypothetical protein | EHI_146120 |
| 67 | hypothetical protein | EHI_191400 |
| 68 | cysteine proteinase, putative | EHI_010850 |
| 70 | hypothetical protein | EHI_020710 |
| 70 | hypothetical protein | EHI_162770 |
| 71 | actin binding protein, putative | EHI_094060 |
| 74 | hypothetical protein | EHI_013080 |
| 74 | ubiquitin, putative | EHI_156660 |
| 77 | regulator of nonsense transcripts, putative | EHI_043440 |
| 77 | regulator of nonsense transcripts, putative | EHI_070810 |
| 77 | regulator of nonsense transcripts, putative | EHI_110840 |
| 77 | regulator of nonsense transcripts, putative | EHI_148970 |
| 77 | regulator of nonsense transcripts, putative | EHI_178520 |
| 77 | regulator of nonsense transcripts, putative | EHI_193520 |
| 78 | Rho guanine nucleotide exchange factor, putative | EHI_021230 |
| 82 | hypothetical protein, conserved domain containing | EHI_016000 |
| 83 | hypothetical protein | EHI_092490 |
| 83 | hypothetical protein, conserved | EHI_181190 |
| 84 | C2 domain containing protein | EHI_059860 |
| 85 | hypothetical protein | EHI_095510 |
| 85 | hypothetical protein | EHI_154760 |
| 86 | 60S ribosomal protein L30, putative | EHI_192800 |
| 87 | AIG1 family protein | EHI_067730 |
| 87 | AIG1 family protein | EHI_089670 |
| 87 | AIG1 family protein | EHI_115160 |
| 87 | AIG1 family protein | EHI_115150 |
| 87 | AIG1 family protein | EHI_129470 |
| 87 | AIG1 family protein | EHI_176280 |
| 87 | AIG1 family protein | EHI_195250 |
| 87 | AIG1 family protein | EHI_199470 |
| 88 | nucleosome assembly protein, putative | EHI_019180 |
| 90 | hypothetical protein | EHI_087210 |
| 90 | hypothetical protein | EHI_188000 |
| 91 | Adapter-related protein complex 3 (AP-3) subunit, putative | EHI_164810 |
| 92 | dual specificity protein phosphatase, putative | EHI_020260 |
| 92 | dual specificity protein phosphatase, putative | EHI_059820 |
| 93 | hypothetical protein | EHI_003270 |
| 93 | hypothetical protein | EHI_003580 |
| 93 | hypothetical protein | EHI_104200 |
| 93 | hypothetical protein | EHI_106360 |
| 93 | hypothetical protein | EHI_131460 |
| 95 | hypothetical protein | EHI_050730 |
| 95 | hypothetical protein | EHI_129900 |
| 95 | hypothetical protein | EHI_159470 |
| 96 | hypothetical protein | EHI_009920 |
| 98 | heat shock protein, putative | EHI_022620 |
| 98 | heat shock protein, putative | EHI_042860 |
| 99 | hypothetical protein | EHI_062550 |
| 100 | hypothetical protein | EHI_020720 |
| 102 | hypothetical protein | EHI_095320 |
| 103 | GTP-binding protein | EHI_031410 |
| 104 | hypothetical protein, conserved | EHI_114220 |
| 104 | hypothetical protein, conserved | EHI_184500 |
| 105 | hypothetical protein | EHI_082090 |
| 105 | syntaxin, putative | EHI_164320 |
| 106 | beta-amylase, putative | EHI_058340 |
| 107 | high mobility group (HMG) box domain containing protein | EHI_087410 |
| 109 | hypothetical protein | EHI_035100 |
| 109 | hypothetical protein | EHI_156220 |
| 111 | hypothetical protein | EHI_020990 |
| 111 | hypothetical protein | EHI_078540 |
| 111 | hypothetical protein | EHI_095300 |
| 111 | hypothetical protein | EHI_154690 |
| 112 | hypothetical protein | EHI_089420 |
| 117 | BRAC1 domain containing protein | EHI_156290 |
| 118 | metallo-beta-lactamase family protein | EHI_012600 |
| 119 | PH domain containing protein kinase, putative | EHI_088110 |
| 120 | PCI domain containing protein | EHI_078210 |
| 121 | hypothetical protein | EHI_074600 |
| 122 | serine/threonine-protein phosphatase, putative | EHI_010290 |
| 123 | endonuclease V, putative | EHI_101390 |
| 124 | cullin family protein | EHI_118180 |
| 125 | heat shock transcription factor, putative | EHI_137000 |
| 126 | hypothetical protein | EHI_097050 |
| 126 | hypothetical protein | EHI_116050 |
| 126 | hypothetical protein | EHI_147990 |
| 127 | U2 snRNP auxiliary factor small subunit, putative | EHI_016140 |
| 128 | vesicle-fusing ATPase, putative | EHI_004640 |
| 129 | phospholipid-transporting P-type ATPase, putative | EHI_024120 |
| 130 | formin homology 2 family protein | EHI_197230 |
| 132 | hypothetical protein | EHI_004550 |
| 132 | TolA-like protein, putative | EHI_052780 |
| 132 | hypothetical protein | EHI_086690 |
| 133 | hypothetical protein | EHI_017900 |
| 133 | hypothetical protein | EHI_057810 |
| 133 | hypothetical protein | EHI_087690 |
| 133 | hypothetical protein | EHI_109270 |
| 133 | hypothetical protein | EHI_135600 |
| 133 | hypothetical protein | EHI_190420 |
| 133 | hypothetical protein | EHI_191520 |
| 135 | hypothetical protein | EHI_148000 |
| 135 | hypothetical protein, pseudogene | EHI_153940 |
| 136 | F-box domain containing protein | EHI_049810 |
| 138 | ABC transporter, putative | EHI_197670 |
| 139 | competence protein ComEC, putative | EHI_125130 |
| 140 | protein kinase domain containing protein | EHI_059040 |
| 140 | protein kinase domain containing protein | EHI_103240 |
| 141 | phospholipase, patatin family protein | EHI_073330 |
| 145 | hypothetical protein | EHI_011560 |
| 145 | hypothetical protein | EHI_065890 |
| 145 | hypothetical protein | EHI_075310 |
| 145 | hypothetical protein | EHI_091350 |
| 145 | hypothetical protein | EHI_183210 |
| 149 | hypothetical protein, conserved domain containing | EHI_056360 |
| 150 | hypothetical protein | EHI_044720 |
| 152 | hypothetical protein | EHI_132850 |
| 155 | Rho guanine nucleotide exchange factor, putative | EHI_006140 |
| 156 | Rho GTPase activating protein, putative | EHI_056450 |
| 157 | galactose-specific adhesin light subunit, putative | EHI_049690 |
| 157 | galactose-inhibitable lectin, putative | EHI_058330 |
| 157 | Gal/GalNAc lectin light subunit | EHI_148790 |
| 157 | galactose-inhibitable lectin 35 kDa subunit precursor, putative | EHI_183400 |
| 159 | hypothetical protein | EHI_094150 |
| 165 | serine palmitoyltransferase, putative | EHI_069310 |
| 165 | alanine aminotransferase, putative | EHI_159710 |
| 166 | eukaryotic translation initiation factor 4E, putative | EHI_013220 |
| 167 | hypothetical protein | EHI_180940 |
| 169 | hypothetical protein, conserved | EHI_095050 |
| 170 | hypothetical protein | EHI_029730 |
| 170 | hypothetical protein, conserved domain containing | EHI_031960 |
| 171 | hypothetical protein | EHI_004440 |
| 171 | hypothetical protein | EHI_012080 |
| 172 | Skp1 family protein | EHI_134960 |
| 173 | leucine rich repeat and phosphatase domain containing protein | EHI_165170 |
| 175 | chitobiosyldiphosphodolichol beta-mannosyltransferase, putative | EHI_028950 |
| 175 | phosphatidylinositol N-acetylglucosaminyltransferase subunit A, putative | EHI_105870 |
| 176 | long-chain-fatty-acid--CoA ligase, putative | EHI_132030 |
| 182 | acetyltransferase, putative | EHI_112790 |
| 183 | hypothetical protein | EHI_004060 |
| 183 | hypothetical protein | EHI_046040 |
| 183 | hypothetical protein | EHI_104230 |
| 188 | elongation factor 2 | EHI_164510 |
| 188 | elongation factor 2 | EHI_166810 |
| 188 | elongation factor 2 | EHI_189490 |
| 196 | hypothetical protein | EHI_194050 |
| 197 | apyrase, putative | EHI_112890 |
| 199 | histidine acid phosphatase family protein | EHI_059910 |
| 200 | grainin, putative | EHI_120360 |
| 207 | 5'-3' exonuclease domain containing protein | EHI_093360 |
| 207 | 5'-3' exonuclease domain containing protein | EHI_133330 |
| 208 | surface antigen ariel1, putative | EHI_098180 |
| 208 | surface antigen ariel1, putative | EHI_131360 |
| 208 | surface antigen ariel1, putative | EHI_172850 |
| 218 | hypothetical protein, conserved | EHI_041580 |
| 218 | hypothetical protein, conserved | EHI_093890 |
| 218 | hypothetical protein, conserved | EHI_098440 |
| 219 | protein kinase, putative | EHI_011170 |
| 224 | 3' exoribonuclease family protein | EHI_188080 |
| 225 | PH domain containing protein | EHI_192960 |
| 227 | Rab GTPase activating protein, putative | EHI_169850 |
| 228 | galactose-specific adhesin 170kD subunit, putative | EHI_042370 |
| 229 | hypothetical protein | EHI_128330 |
| 230 | type A flavoprotein, putative | EHI_064530 |
| 230 | type A flavoprotein, putative | EHI_152650 |
| 232 | NADPH-dependent FMN reductase domain containing protein | EHI_022600 |
| 232 | NADPH-dependent FMN reductase domain containing protein | EHI_103260 |
| 232 | NADPH-dependent FMN reductase domain containing protein | EHI_181710 |
| 236 | cysteine protease, putative | EHI_084060 |
| 236 | cysteine protease, putative | EHI_121160 |
| 236 | cysteine protease, putative | EHI_160330 |
| 236 | cysteine protease, putative | EHI_179600 |
| 237 | hypothetical protein | EHI_029500 |
| 238 | hypothetical protein | EHI_014510 |
| 238 | hypothetical protein | EHI_038030 |
| 238 | hypothetical protein | EHI_074940 |
| 238 | hypothetical protein | EHI_174630 |
| 238 | hypothetical protein | EHI_174380 |
| 244 | hypothetical protein | EHI_059340 |
| 246 | chitinase, putative | EHI_092100 |
| 247 | kinase, PfkB family | EHI_157120 |
| 253 | exonuclease, putative | EHI_094260 |
| 256 | syntaxin, putative | EHI_080870 |
| 259 | TBC/Rab GTPase activating domain containing protein | EHI_045210 |
| 259 | TBC/Rab GTPase activating domain containing protein | EHI_052170 |
| 264 | hypothetical protein | EHI_074490 |
| 264 | hypothetical protein | EHI_145490 |
| 266 | inositol hexakisphosphate kinase, putative | EHI_106120 |
| 267 | 60S ribosome subunit biogenesis protein NIP7, putative | EHI_017890 |
| 267 | 60S ribosome subunit biogenesis protein NIP7, putative | EHI_031350 |
| 267 | 60S ribosome subunit biogenesis protein NIP7, putative | EHI_135590 |
| 269 | hypothetical protein | EHI_102050 |
| 274 | AIG1 family protein | EHI_022500 |
| 274 | AIG1 family protein | EHI_144270 |
| 277 | hypothetical protein | EHI_018000 |
| 277 | hypothetical protein | EHI_041780 |
| 279 | calcineurin catalytic subunit A, putative | EHI_118600 |
| 282 | hypothetical protein | EHI_185400 |
| 283 | hypothetical protein | EHI_096370 |
| 284 | 60S ribosomal protein L7, putative | EHI_025830 |
| 286 | hypothetical protein | EHI_014910 |
| 288 | nuclear complex protein 4, putative | EHI_083570 |
| 288 | ccaat-box-binding transcription factor, putative | EHI_132370 |
| 294 | serine-rich protein | EHI_116360 |
| 294 | surface antigen ariel1, putative | EHI_186470 |
| 301 | peptidase S54 (rhomboid) family protein | EHI_060330 |
| 302 | coatomer protein gamma subunit, putative | EHI_040700 |
| 302 | AP-2 complex protein, putative | EHI_045710 |
| 303 | serine acetyltransferase 1 | EHI_021570 |
| 306 | hypothetical protein | EHI_075730 |
| 306 | hypothetical protein | EHI_075740 |
| 309 | hypothetical protein | EHI_001750 |
| 309 | hypothetical protein | EHI_020860 |
| 309 | hypothetical protein | EHI_020870 |
| 309 | hypothetical protein | EHI_060010 |
| 311 | S1 RNA binding domain-containing protein | EHI_166590 |
| 315 | Ser/Thr protein phosphatase, putative | EHI_017600 |
| 321 | serine-threonine-isoleucine rich protein, putative | EHI_073630 |
| 322 | 26S protease regulatory subunit, putative | EHI_185410 |
| 322 | 26S protease regulatory subunit, putative | EHI_187600 |
| 323 | chaperonin 1 60 kDa | EHI_178570 |
| 324 | long-chain-fatty-acid--CoA ligase, putative | EHI_025090 |
| 326 | hypothetical protein | EHI_008420 |
| 332 | ribosomal protein L27a, putative | EHI_020370 |
| 335 | hypothetical protein, conserved | EHI_052040 |
| 335 | hypothetical protein | EHI_178460 |
| 342 | magnesium and cobalt transport protein CorA, putative | EHI_175500 |
| 343 | 60S ribosomal protein L34 | EHI_167050 |
| 346 | hypothetical protein | EHI_028220 |
| 346 | hypothetical protein | EHI_076780 |
| 346 | hypothetical protein | EHI_113200 |
| 348 | transcription factor S-II (TFIIS) domain protein | EHI_055430 |
| 350 | hypothetical protein | EHI_096350 |
| 351 | hypothetical protein | EHI_056350 |
| 351 | hypothetical protein | EHI_133910 |
| 352 | mutT/nudix family protein | EHI_054150 |
| 354 | tRNA (cytosine-5-)-methyltransferase, putative | EHI_140970 |
| 358 | nucleoside transporter, putative | EHI_110730 |
| 359 | hypothetical protein | EHI_123120 |
| 363 | hypothetical protein | EHI_168830 |
| 367 | 60S ribosomal protein L2/L8, putative | EHI_127200 |
| 370 | serine/threonine-protein kinase, putative | EHI_148440 |
| 374 | hypothetical protein | EHI_127460 |
| 380 | elongation factor 1-alpha 1 | EHI_011210 |
| 380 | elongation factor 1-alpha 1 | EHI_052400 |
| 380 | elongation factor 1-alpha 1 | EHI_102170 |
| 381 | Legume-like lectin family protein, membrane-bound | EHI_104370 |
| 382 | hypothetical protein | EHI_198440 |
| 384 | hypothetical protein | EHI_106800 |
| 386 | 60S ribosomal protein L44, putative | EHI_153070 |
| 389 | hypothetical protein | EHI_110830 |
| 390 | hypothetical protein | EHI_070800 |
| 406 | DNA polymerase, putative | EHI_132860 |
| 406 | DNA polymerase, putative | EHI_164190 |
| 410 | hypothetical protein | EHI_060210 |
| 410 | hypothetical protein | EHI_140870 |
| 410 | hypothetical protein | EHI_156910 |
| 417 | inositol polyphosphate-5-phosphatase, putative | EHI_046590 |
| 418 | hypothetical protein | EHI_016900 |
| 418 | hypothetical protein | EHI_132250 |
| 421 | hypothetical protein, conserved | EHI_121360 |
| 424 | 40S ribosomal protein S15, putative | EHI_046690 |
| 424 | 40S ribosomal protein S15, putative | EHI_064570 |
| 426 | lecithin:cholesterol acyltransferase, putative | EHI_099180 |
| 427 | metallo-beta-lactamase superfamily protein | EHI_115720 |
| 431 | hypothetical protein | EHI_056090 |
| 448 | hypothetical protein | EHI_117690 |
| 448 | hypothetical protein | EHI_159660 |
| 461 | hydrolase, carbon-nitrogen family | EHI_035680 |
| 462 | metal cation transporter, zinc (Zn2+)-iron (Fe2+) permease (ZIP) | EHI_152760 |
| 470 | hypothetical protein | EHI_172280 |
| 470 | leukocyte-endothelial cell adhesion molecule 3, putative | EHI_201200 |
| 472 | hypothetical protein | EHI_170190 |
| 473 | glutamate synthase beta subunit, putative | EHI_110520 |
| 476 | copine, putative | EHI_140260 |
| 480 | hypothetical protein | EHI_017550 |
| 481 | hypothetical protein, conserved | EHI_030770 |
| 484 | Sir2 family transcriptional regulator, putative | EHI_007500 |
| 485 | hypothetical protein | EHI_155790 |
| 487 | heat shock protein 90, putative | EHI_196940 |
| 489 | 60S ribosomal protein L36, putative | EHI_199940 |
| 493 | hypothetical protein | EHI_074480 |
| 493 | hypothetical protein | EHI_145500 |
| 497 | hypothetical protein | EHI_132870 |
| 497 | hypothetical protein | EHI_196790 |
| 501 | hypothetical protein | EHI_017670 |
| 503 | hypothetical protein | EHI_010380 |
| 504 | hypothetical protein | EHI_022270 |
| 504 | hypothetical protein | EHI_192040 |
| 509 | Phosphatidylinositol-4,5-bisphosphate 3-kinase catalytic subunit, putative | EHI_091940 |
| 513 | hypothetical protein | EHI_114200 |
| 514 | hypothetical protein | EHI_184220 |
| 516 | ethanolamine phosphotransferase, putative | EHI_055140 |
| 521 | surface antigen ariel1, putative | EHI_036490 |
| 521 | dextranase precursor, putative | EHI_182460 |
| 527 | hypothetical protein | EHI_090370 |
| 532 | hypothetical protein, conserved | EHI_189410 |
| 539 | amino acid-polyamine transporter, putative | EHI_020320 |
| 542 | hypothetical protein, conserved | EHI_131910 |
| 543 | F-box domain containing protein, putative | EHI_103710 |
| 546 | protein transport protein Sec24, putative | EHI_048310 |
| 553 | protein farnesyltransferase beta subunit, putative | EHI_006190 |
| 553 | Rab geranylgeranyltransferase beta subunit, putative | EHI_025110 |
| 566 | hypothetical protein, conserved | EHI_097750 |
| 568 | hypothetical protein | EHI_004540 |
| 571 | hypothetical protein | EHI_078580 |
| 578 | iron-sulfur flavoprotein, putative, pseudogene | EHI_164520 |
| 582 | P-glycoprotein 6, putative | EHI_081130 |
| 582 | multidrug resistance protein, putative | EHI_100320 |
| 586 | pore-forming peptide ameobapore A precursor, putative | EHI_159480 |
| 588 | helicase, putative | EHI_090040 |
| 589 | hypothetical protein | EHI_150650 |
| 590 | hypothetical protein | EHI_066300 |
| 594 | hypothetical protein | EHI_013860 |
| 598 | Thioredoxin domain-containing protein 2, putative | EHI_190880 |
| 601 | Plasma membrane calcium-transporting ATPase, putative | EHI_016480 |
| 603 | vacuolar sorting protein 9 (VPS9) domain containing protein | EHI_127450 |
| 604 | hypothetical protein, conserved | EHI_166260 |
| 609 | methionine gamma-lyase | EHI_144610 |
| 613 | MmpL efflux pump, putative | EHI_000920 |
| 615 | hypothetical protein | EHI_006670 |
| 626 | 60S ribosomal protein L37a, putative | EHI_148230 |
| 627 | zinc finger protein, putative | EHI_176800 |
| 630 | ARP2/3 complex 34 kda subunit, putative | EHI_091250 |
| 637 | hypothetical protein | EHI_151450 |
| 638 | SH2-protein kinase domain containing protein | EHI_055990 |
| 638 | SH2-protein kinase domain containing protein | EHI_128700 |
| 639 | dihydrouridine synthase (Dus) family protein | EHI_164780 |
| 640 | maltose O-acetyltransferase, putative | EHI_157020 |
| 649 | hypothetical protein, conserved | EHI_019970 |
| 656 | 3'(2'),5'-bisphosphate nucleotidase, putative | EHI_179820 |
| 660 | hypothetical protein | EHI_016130 |
| 664 | hypothetical protein | EHI_132040 |
| 681 | cylicin-2, putative | EHI_090640 |
| 682 | tRNA (guanine-N(1)-)-methyltransferase TRM10 | EHI_168400 |
| 692 | hypothetical protein, conserved | EHI_197660 |
| 693 | vacuolar proton ATPase subunit, putative | EHI_107280 |
| 694 | N-ethylmaleimide-sensitive factor attachment protein, alpha, putative | EHI_000300 |
| 705 | replication factor A protein 1, putative | EHI_062980 |
| 707 | F-actin capping protein beta subunit, putative | EHI_005020 |
| 707 | F-actin capping protein subunit beta, putative | EHI_134490 |
| 708 | DEAD/DEAH box helicase, putative | EHI_036900 |
| 743 | protein kinase, putative | EHI_075540 |
| 746 | ariadne-1, putative | EHI_080850 |
| 746 | ariadne-1, putative | EHI_159910 |
| 754 | mucin-like protein 1 precursor, putative | EHI_066970 |
| 756 | sodium/proton antiporter, putative | EHI_176180 |
| 758 | hypothetical protein, conserved | EHI_059640 |
| 760 | RNA-binding protein, putative | EHI_033350 |
| 762 | DNA mismatch repair protein Msh2, putative | EHI_172750 |
| 764 | CDP-diacylglycerol--inositol3-phosphatidyltransferase, putative | EHI_069630 |
| 769 | protein kinase, putative | EHI_011510 |
| 770 | 60S ribosomal protein L27, putative | EHI_146370 |
| 772 | hypothetical protein | EHI_154190 |
| 773 | hypothetical protein | EHI_074380 |
| 774 | cysteine protease, putative | EHI_123950 |
| 784 | Der1 family protein, putative | EHI_035580 |
| 787 | snoRNA binding protein, putative | EHI_183900 |
| 796 | L-myo-inositol-1-phosphate synthase, putative | EHI_165270 |
| 798 | hypothetical protein, conserved | EHI_114940 |
| 801 | dipeptidyl-peptidase, putative | EHI_133430 |
| 819 | retroviral aspartyl protease domain-containing protein | EHI_015550 |
| 823 | purine nucleoside phosphorylase, putative | EHI_012720 |
| 825 | deoxyuridine 5'-triphosphate nucleotidohydrolase, putative | EHI_189420 |
| 828 | aspartyl aminopeptidase, putative | EHI_106690 |
| 833 | Rab GTPase activating protein, putative | EHI_149900 |
| 834 | 60S ribosomal protein L19, putative | EHI_096850 |
| 837 | oligopeptidase A, putative | EHI_185560 |
| 839 | hypothetical protein | EHI_197880 |
| 846 | peptidyl-prolyl cis-trans isomerase, putative | EHI_044730 |
| 849 | T-complex protein 1 subunit eta, putative | EHI_083260 |
| 854 | hypothetical protein | EHI_027740 |
| 861 | protein kinase, putative | EHI_128070 |
| 867 | melanocyte prolifeating gene 1, putative | EHI_012050 |
| 868 | protein phosphatase, putative | EHI_114170 |
| 873 | hypothetical protein, conserved | EHI_000700 |
| 883 | 40S ribosomal protein S19, putative | EHI_146570 |
| 885 | multidrug resistance-associated protein, putative | EHI_084730 |
| 888 | hypothetical protein, conserved | EHI_198970 |
| 889 | hypothetical protein | EHI_134850 |
| 890 | hypothetical protein | EHI_189170 |
| 891 | hypothetical protein, conserved | EHI_135140 |
| 892 | P-glycoprotein 5, putative | EHI_125030 |
| 894 | hypothetical protein | EHI_024890 |
