# Supplementary material for: New Assembly, Reannotation and Analysis of the Entamoeba histolytica Genome Reveal New Genomic Features and Protein Content Information
Source: PLoS Negl Trop Dis. 2010 Jun 15;4(6):e716. doi: 10.1371/journal.pntd.0000716 (PMC2886108; doi:10.1371/journal.pntd.0000716)
Supplement: Text S1 — Assembly supplement. (0.05 MB DOC) [file pntd.0000716.s006.doc]

**Assembly Supplement**

**Manuscript:**

New assembly, reannotation and analysis of the Entamoeba histolytica genome reveal new genomic features and protein content information. Lorenzi et al. 2010.

**Supplemental Text**

A total of 643,948 reads were obtained directly from the Sanger Institute and JCVI databases. All but 58,438 reads were paired. Disappointing assembly results led to scrutiny and filtering of the reads. First, the reads were used to reconstruct and confirm a previously assembled *E. histolytica* plasmid sequence [1]. The reconstructed sequence was used to identify and exclude plasmid-like reads from the genome assembly. A read was excluded if it, or its mate pair, displayed 95% identity, over 90% of the read clear range as aligned by MUMmer [2], to the plasmid. The plasmid filter excluded 116,661 reads. Second, 75,314 reads were excluded for sequence similarity, by the same alignment criteria, to *E. histolytica* tRNA cassettes [3]. Of these, 80% had their mate also excluded by the filter. Third, 1040 reads were excluded for having high coverage in K-mers whose frequency was high in the overall data set. Of these filtered reads, many contained repeats of the single letter C.

The reads were trimmed with UMD Overlapper [4] to determine a clear range for every read. The trimmed reads were assembled with Celera Assembler version 3.10 [5]. The source code is available and tagged as VERSION-3_10 in cvs on Source Forge (http://wgs-assembler.sf.net). The following non-standard assembly options were used. The meryl K-mer frequency limit was set to 1000 (default=100) to allow more repetitive regions to seed overlaps. The assumed error rate for building unitigs was set to 0.5% (default=1.5%) to separate similar repeats. The genome size (default=none) was set to 10Mbp to reduce sensitivity to coverage-based repeat detection. Without this setting, the assembler would have derived its own coverage-based value of 21,267,282 bases. The OBT module was not activated. The assembly ran on AMD Opteron processors having 64GB RAM and the Suse 10.1 Linux operating system.

The assembler produced 2173 contigs in 1530 scaffolds. Total scaffold span (including estimated gap sizes) was 21,199,346 bases. There were 20,772,429 bases in scaffolds (excluding gaps). The average intra-scaffold gap size estimate was 664 bases. The scaffold N50 statistic was 49,018 and the contig N50 was 29,517 bases. Of the 442,379 reads that passed the assembler’s quality filter, 87% were assembled into contigs and 73% into contigs of at least10Kbp. Not all contigs passed the downstream annotation quality filters.

Assembly correctness was explored by visual inspection [6]. Analysis of the 50 largest scaffolds revealed one possible collapse of a nearly identical 2-copy tandem repeat but no serious problems. Scaffold ends seemed enriched for repetitive sequence. Of the 100 scaffold ends examined, 57% were composed of a repeat and 19% had a mate link into a repeat outside the scaffold. Repeat was operationally defined as a surrogate unitig in the Celera Assembler output, meaning a high-coverage contig used at least once in scaffolds.

**Supplemental Acknowledgements**

The authors are grateful to Michael Schatz, Art Delcher, and Hean Koo for analysis of the reads, to Prakriti Mudvari for inspection of the assembly, and to Granger Sutton, Brian Walenz, Steven Salzberg, Mihai Pop, Martin Shumway, James Yorke, Aleksey Zimin, and Mike Roberts for helpful discussions.

**Supplemental References**

1. Sehgal, D., et al., *Nucleotide sequence organisation and analysis of the nuclear ribosomal DNA circle of the protozoan parasite Entamoeba histolytica.* Mol Biochem Parasitol, 1994. **67**(2): p. 205-14.

2. Kurtz, S., et al., *Versatile and open software for comparing large genomes.* Genome Biol, 2004. **5**(2): p. R12.

3. Clark, C.G., et al., *Unique organisation of tRNA genes in Entamoeba histolytica.* Mol Biochem Parasitol, 2006. **146**(1): p. 24-9.

4. Roberts, M., et al., *A preprocessor for shotgun assembly of large genomes.* J Comput Biol, 2004. **11**(4): p. 734-52.

5. Myers, E.W., et al., *A whole-genome assembly of Drosophila.* Science, 2000. **287**(5461): p. 2196-204.

6. Schatz, M.C., et al., *Hawkeye: an interactive visual analytics tool for genome assemblies.* Genome Biol, 2007. **8**(3): p. R34.
